# CLEAPA: a framework for exploring the conformational landscape of cryo-EM using energy-aware pathfinding algorithm

**DOI:** 10.1093/bioinformatics/btae345

**Published:** 2024-06-05

**Authors:** Teng-Yu Lin, Szu-Chi Chung

**Affiliations:** Department of Applied Mathematics, National Sun Yat-sen University, Kaohsiung 804, Taiwan; Department of Applied Mathematics, National Sun Yat-sen University, Kaohsiung 804, Taiwan

## Abstract

**Motivation:**

Cryo-electron microscopy (cryo-EM) is a powerful technique for studying macromolecules and holds the potential for identifying kinetically preferred transition sequences between conformational states. Typically, these sequences are explored within two-dimensional energy landscapes. However, due to the complexity of biomolecules, representing conformational changes in two dimensions can be challenging. Recent advancements in reconstruction models have successfully extracted structural heterogeneity from cryo-EM images using higher-dimension latent space. Nonetheless, creating high-dimensional conformational landscapes in the latent space and then searching for preferred paths continues to be a formidable task.

**Results:**

This study introduces an innovative framework for identifying preferred trajectories within high-dimensional conformational landscapes. Our method encompasses the search for the minimum energy path in the graph, where edge weights are determined based on the energy estimation at each node using local density. The effectiveness of this approach is demonstrated by identifying accurate transition states in both synthetic and real-world datasets featuring continuous conformational changes.

**Availability and implementation:**

The CLEAPA package is available at https://github.com/tengyulin/energy_aware_pathfinding/.

## 1 Introduction

Cryo-electron microscopy (cryo-EM) involves imaging biological samples in a nearly native frozen-hydrated state at cryogenic temperatures using an electron microscope. Contrary to the well-established methods of X-ray crystallography and nuclear magnetic resonance, which predominantly determine single static macromolecular structures, cryo-EM has the capability to reconstruct 3D volumes for discrete conformations. This ability positions it as a powerful tool for analyzing the structures of macromolecular machines during dynamic processes.

Recent advances in cryo-EM reconstruction models have effectively extracted structural heterogeneity across a continuous spectrum Toader *et al.* (2023), thereby enhancing their capacity to analyze complex molecular structures. Typically, these models are based on methodologies such as auto-encoders (Zhong *et al.* 2021, Punjani and Fleet 2023), eigen-analysis (Punjani and Fleet 2021), or manifold learning (Dashti *et al.* 2014, 2020). In practice, the latent spaces derived from these models effectively capture the conformational landscape, indicating their potential to elucidate protein functionality. Specifically, identifying a trajectory that bridges initial and final states within conformational landscapes—traversing the path of least cumulative energy—becomes critical in understanding the function of proteins. This least energy path can be found by employing pathfinding algorithms (Seitz and Frank 2020, Zhong *et al.* 2021, Wu *et al.* 2022) on the landscape. The use of pathfinding algorithms has demonstrated the potential to significantly expedite the laborious trajectory-finding workflow, which may take days to weeks to complete Davis *et al.* (2016). Additionally, these algorithms can efficiently generate movies by sampling along the latent space, providing a crucial reference for biologists to understand protein functions Zhong *et al.* (2021).

Despite the promise of these advancements, a key question pertains to the accuracy with which computational tools can identify functionally relevant conformational trajectories. The situation is worsened since there is no standard to compare the dynamic movies that describe conformation changes, which currently require further analysis by biologists using laborious experimental techniques (Supplementary Appendix A.2). To bridge this gap and focus on the algorithm itself, we introduce a comprehensive synthetic generation workflow for continuous conformational changes in this study. Additionally, we propose innovative metrics for assessing the effectiveness of pathfinding algorithms. This benchmarking is crucial not only for understanding the strengths and limitations of existing methods but also for fostering further development and validation of these techniques.

Another issue is that current pathfinding algorithms either overlook local density considerations or cannot operate efficiently in high-dimensional spaces. To address this, we propose an algorithm that navigates the typically high-dimensional latent space to identify the most probable path. This approach deviates from traditional minimum energy path (MEP) algorithms (Seitz and Frank 2020, Wu *et al.* 2022), which generally search in a two-dimensional energy landscape. Instead, our method directly constructs nearest neighbor graphs within the latent space. Additionally, we introduce a novel statistic that estimates local density at each node and assigns graph edge weights as energy-aware values based on the Boltzmann factor. Our search process circumvents the need for a 2D MEP algorithm by leveraging the full potential of the high-dimensional latent space, thereby enabling the study of more complex systems. Experimental results indicate that our algorithm significantly outperforms conventional methods in both synthetic and real-world datasets. Finally, we have organized our framework into a modular package, CLEAPA, designed for easy integration and use by the cryo-EM community.

## 2 Methods

### 2.1 CLEAPA: the proposed conformation analysis framework

**Figure 1.**
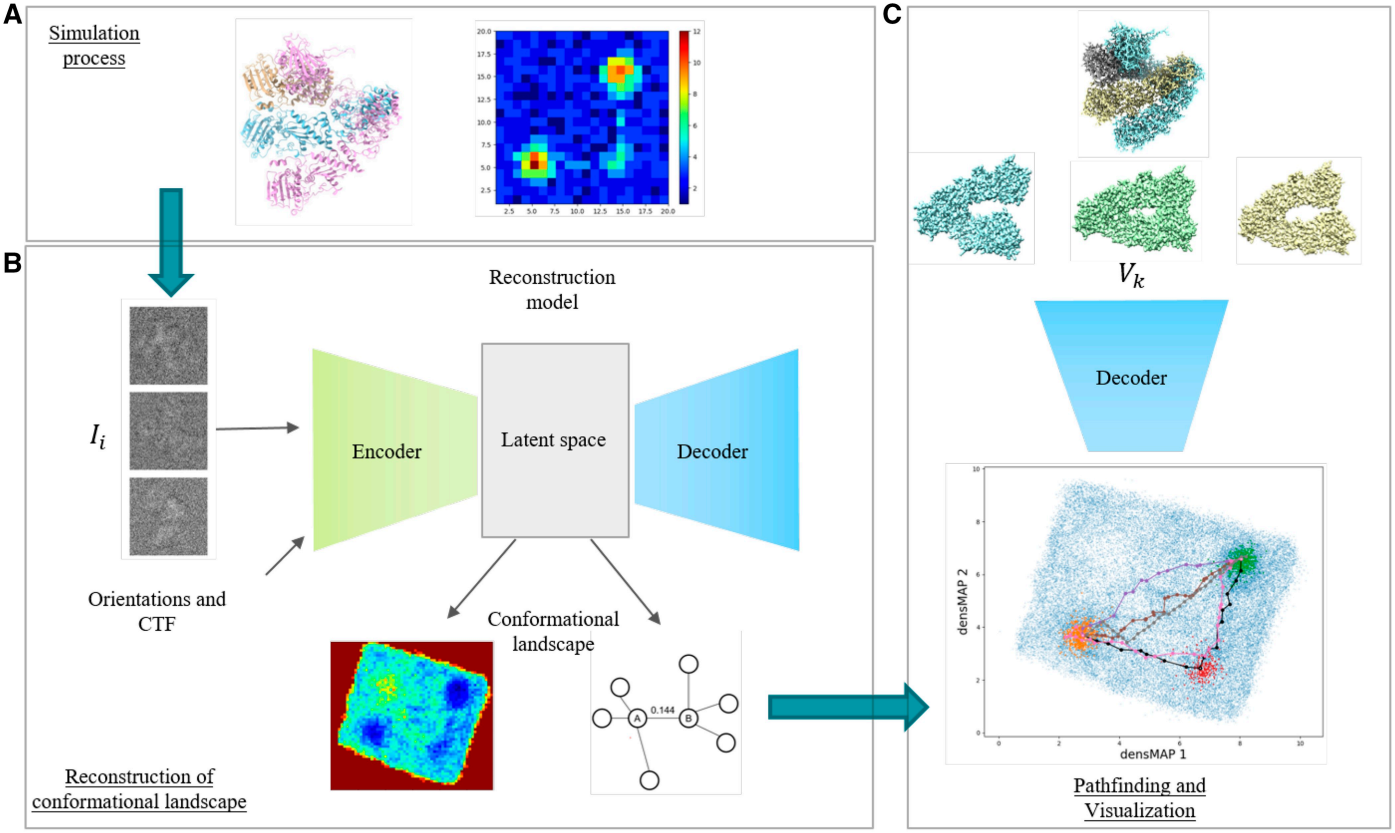
CLEAPA framework: the proposed framework for conformation analysis in cryo-EM. (**A**): Novel simulation process for continuous conformational changes. (**B**): The reconstruction of the underlying conformational landscape and the generation of heterogeneity representation. (**C**): Pathfinding, visualization, and generation of the 3D density map.

Our proposed conformation analysis pipeline is illustrated in Fig. 1. The simulation process efficiently generates a conformational landscape using the atomic model and the designed occupancy map, as depicted in Fig. 1A. To reconstruct the conformational landscape of the dataset, one can input either a synthetic dataset or real experimental data into the reconstruction model. In the latter case, the orientations and Contrast Transfer Function parameters are determined by upstream software, such as RELION (Scheres 2012). The reconstruction model then identifies a low-dimensional (e.g. 8–10) latent representation of the underlying conformational landscape (Fig. 1B, upper). Once the conformational landscape has been reconstructed, two approaches are employed to identify paths using the latent space. The first involves using a density-aware dimension reduction method and a 2D histogram (Fig. 1B, lower-left) to construct a 2D conformational landscape and apply the 2D MEP algorithm. The second approach constructs a nearest neighbor graph with energy-aware edges within the latent space (Fig. 1B, lower-right) and employs graph traversal algorithms to delineate preferred trajectories between states. The resulting paths are visualized in two-dimensional space using the same density-aware dimension reduction method (Fig. 1C, bottom). Additionally, the veracity and accuracy of these paths are assessed using our proposed metrics. Finally, points along the trajectory can be sampled, particularly focusing on areas of higher local density that represent stable states. These points are input into the decoder, generating 3D density maps of various major states or a movie illustrating the conformational changes in 3D space (Fig. 1C, upper).

### 2.2 Simulation process for continuous conformational changes

Benchmarking pathfinding algorithms in cryo-EM represents a novel topic. To address this challenge, we devised a synthetic process inspired by Seitz *et al.* (2019) to simulate continuous conformational changes using occupancy maps. The synthesis of datasets for our experiment entails five steps (Supplementary Fig. 13). Initially, we generate a series of conformational states using atomic models. We induce these varying states by applying rotations and movements to different chains within the atomic model, thus emulating the natural dynamics of molecules. Subsequently, we translate these atomic models into 3D density maps using EMAN2 (Tang *et al.* 2007). Third, we apply an occupancy map to the 3D volumes generated in the second step. The occupancy map indicates the prevalence of each conformational state, designed to emulate the cryo-EM imaging process where microscopes more frequently capture stable states. To create this map (Supplementary Fig. 14), we first generate data points in the same dimensionality as our chosen degrees of conformational changes using a Gaussian mixture model. This method allows us to represent stable states as clusters, rather than single points, leading to a more continuous landscape akin to actual energy landscapes. We then segment the space into areas corresponding to different conformational changes. In the fourth step, we use RELION to project each volume into 2D images using the forward process of cryo-EM.Finally, we adjust the signal-to-noise ratio to 0.1.

### 2.3 Building and visualizing the 2D conformational landscape

In practice, constructing a high-dimensional landscape is challenging due to the lack of efficient approaches, leading to pathfinding typically being conducted in 2D space. While tools like Uniform Manifold Approximation and Projection (UMAP) (McInnes *et al.* 2018) have proven effective in building energy landscapes (Wu *et al.* 2022) and visualizing heterogeneity (Zhong *et al.* 2021), they do not adequately represent the local density of data points in the original space. This limitation reduces their effectiveness in analyzing conformation mixtures (see Supplementary Appendix A.2). In this study, we introduce the density-preserving manifold embedding method (densMAP; Narayan *et al.* 2021) (see Supplementary Appendix A.3) on the latent space. We found that densMAP offers a more faithful representation of the underlying structure while maintaining the performance of UMAP, as evidenced in Supplementary Fig. 2 and Supplementary Appendix C. It effectively visualizes high-dimensional structural heterogeneity and the paths identified by various pathfinding algorithms, providing deeper insights into the conformational landscape of the dataset. Building on the densMAP, we designed an algorithm that constructs a 2D conformational landscape using a histogram approach and employs POLARIS (Seitz and Frank 2020) to find the preferred trajectories, which we term 2D MEP (see Supplementary Appendix B, Algorithm 2).

### 2.4 Propose energy-aware pathfinding algorithm

To address the challenges associated with identifying the most energetically favorable transition pathways between molecular conformations directly from the latent space, our proposed pathfinding approach consists of three main steps: constructing a graph, determining a threshold for energy estimation, and searching for the MEP. First, we construct a nearest neighbor graph from the latent encodings by treating each data point in the latent space as a graph node. Neighbors are determined based on the Euclidean distance between nodes. The number of neighbors, *N*, serves as a parameter and dictates the range of energy differences within the landscape, with maximum occupancy defined by the maximum number of neighbors for a node. This parameter is relatively insensitive; hence, we set it to a fixed value of *N *=* *50 to balance computational cost and the adequacy of energy differences in identifying accurate paths.

Algorithm 1 Proposed energy-aware pathfinding algorithm

**Require:** Latent space *z*, two points *A* and *B* in the latent space,   *N*, a list of *θ*, (*r_l_*, *r_u_*)

**Ensure:**Best_MEP

1: Construct an *N* nearest neighbor graph *G*(*V*, *E*) from *z*, where neighbors are determined based on Euclidean distance.

2: **for** each *θ* **do**

3:  Utilize Equation 1 to estimate *n_v_* and then convert *n_v_* to energy *E_v_* using Equation 2. Assign the weights of each edge as the average energy between the two connected nodes.

4:  Calculate *r*_0_ (Supplementary Appendix D).

5:  **if**$r_{l}\leq r_{0}\leq r_{u}$**then**

6:   Search for the shortest path between *A* and *B* on *G*(*V*, *E*) using Dijkstra’s algorithm and store it as *MEP*.

7:   Store the path in a list: paths←MEP

8:  **end if**

9: **end for**

10: Determine Best_MEP in *paths* based on the average distance between each node and its neighbors (Equation (S.5))

The second step is the most crucial, where we select a threshold *θ* to estimate the local density for each node, thereby defining the size of the topological grids on the graph (Supplementary Fig. 15). We assess the neighbors of each node against *θ*; those exceeding the threshold are considered distant, potentially belonging to another state, and are thus excluded from calculations. The local density estimation for each node is the count of neighbors within *θ*:

(1) $$n_{v}=\sum_{v\prime \in N(v)}1_{\left\{\left\|v-v\prime \right\|\leq \theta \right\}}$$

where $1_{\left\{\cdot \right\}}$ is the indicator function, *N*(*v*) is the neighbors of node *v*, and ‖·‖ denotes Euclidean norm. We transform *n_v_* into energy, aligned with energy landscape concepts (Fischer *et al.* 2010):

(2) $$\frac{n_{v}}{n_{j}}=\text{exp}(-E_{v}/K_{B}T);\;E_{v}=-\text{log}(\frac{n_{v}}{n_{j}})K_{B}T.$$

Here, *n_j_* is the local density of reference node *j*, which possesses the lowest density estimate across the entire graph, *K_B_* is the Boltzmann constant, and *T* stands for temperature. The edge weights are calculated as the average energy between two nodes. This approach provides a computationally efficient method to approximate local density in high-dimensional spaces using the space defined by the nearest neighbor graph. The selection of *θ* modulates the overall shape and smoothness of the conformational landscape. To accurately portray the landscape, we propose a novel quantile search procedure for *θ*, as described in Supplementary Appendix D.

Finally, we employ Dijkstra’s Algorithm (Cormen *et al.* 2022) to search for the trajectories. With non-negative edge weights defined by average energy, the algorithm efficiently finds the shortest path within the graph. By estimating local density and defining edge weights as energy estimation, the shortest path obtained corresponds to the MEP, representing the most energetically favorable transition pathway between molecular conformations. A summary of our algorithm is detailed in Algorithm 1.

Our pathfinding algorithm offers several advantages. First, it integrates energy information and operates directly within a high-dimensional latent space, eliminating the need for dimension reduction methods. Second, the parameters of our algorithm have intuitive meanings: the parameter *N* determines the total number of energy levels in the landscape, whereas *θ* controls its overall shape. Empirically, this intuitive understanding facilitates easier determination and adjustment of parameters, simplifying the process compared to the hyperparameter tuning required by previous algorithms (see Supplementary Appendix D). Finally, by applying energy-aware weights, we ensure that our algorithm outputs the least energy path, which is more plausible than traditional graph traversal methods that use Euclidean weighting.

### 2.5 Proposed metrics for evaluating pathfinding algorithms

In previous works (Punjani and Fleet 2021, Zhong *et al.* 2021), the output trajectories are often presented as movies, which require further analysis by biologists. The challenge is that directly comparing two movies is computationally expensive, and no standard approach exists. To offer a more objective way to compare different trajectories, we use the predicted path in the latent space and the snapshot at the main state as proxies and provide two categories of metrics. The first category focuses on the alignment between the path generated by an algorithm and the ground truth path. The primary objective of pathfinding algorithms is to identify possible intermediate transition states between two major states. Hence, for each result produced by the algorithm, represented as $P=\{p_{1},\ldots ,p_{q}\}$, which is a sequence of data points in the latent space, we locate the point closest to the center point *g* of the transition state. This center point represents the main transition state we aim to uncover. The Euclidean distance between this closest point and the transition center in the latent space serves as a measure of how accurately each algorithm can identify the transition states:

(3) $$d_{\text{Transition}}(g,P)=\min\left\|g-p_{i}\right\|,\;i=1,\ldots ,q$$

The second measure of closeness considers that the transition process typically consists of a sequence of intermediate states beyond the important transition state, denoted as a sequence $G=\{g_{1},\ldots ,g_{k}\}$. For each state center in this sequence, excluding the starting and ending states, we identify the nearest point on the algorithm-derived path. We then calculate the distance between this point and the state in latent space. The average of these distances across all states provides a summary measure:

(4) $$D_{\text{ave}}(G,P)=\frac{1}{k-2}\sum_{j=2}^{k-1}\min\left\|g_{j}-p_{i}\right\|,\;i=1,\ldots ,q$$

The third distance measure is the Hausdorff distance (Huttenlocher *et al.* 1993). Given the ground truth center of the state sequence *G* and the predicted sequence *P*, the metric is defined as:

(5) $$\begin{array}{cc} D_{\text{worst}}(G,P) & =\max(h(G,P),h(P,G))\\ h(G,P) & =\underset{g\in G}{\max}\underset{p\in P}{\min}\left\|g-p\right\| \end{array}$$

This distance metric provides a worst-case scenario of the maximum discrepancy between two paths.

In the second category of metrics, we focus on comparing the predicted volumes with the transition states. We select the point on the predicted path closest to the center of the transition state in the latent space and use the decoder to generate 3D volumes. Then, we calculate the Fourier Shell Correlation (FSC) (Rosenthal and Henderson 2003), the standard resolution measure in cryo-EM, with the ground truth volume used in the simulation process. The differences between conformational states can usually be captured at medium frequencies in the Fourier domain. Thus, we utilize a frequency of $0.2{Å}^{-1}$ (corresponding to a resolution of 5Å) to compare the correlation with the ground truth volume. The metric that measures the FSC of volumes generated by the closest point on the predicted path *P* and the center of the transition state *g* at 5 Å is defined as:

(6) $$FSC_{5Å}(g,P)=\frac{\sum_{s\in S_{0.2}}{\hat{U}}_{s}^{g}{\hat{V}}_{s}^{P}}{\sqrt{\left(\sum_{s\in S_{0.2}}|{\hat{U}}_{s}^{g}{|}^{2})(\sum_{s\in S_{0.2}}|{\hat{V}}_{s}^{P}{|}^{2}\right)}}$$

Here, ${\hat{U}}_{s}^{g}$ and ${\hat{V}}_{s}^{P}$ represent the frequency components of 3D volumes at the closest point to the center *g* and the path *P*, respectively. $S_{0.2}$ represents the set of voxels in a spherical shell at a distance of $0.2{Å}^{-1}$ from the origin.

## 3 Experiments and results

In this study, we selected cryoDRGN (Zhong *et al.* 2021) as our reconstruction model due to its high precision in recovering the conformational landscape (Supplementary Appendix E). We utilized the centers of major states in the latent space as inputs for the pathfinding algorithms and established the ground truth trajectory by connecting the centers of transition states. Our objective was to compare four pathfinding algorithms capable of directly identifying paths in high-dimensional space. These algorithms include the Graph Traversal algorithm (GT-o) (Zhong *et al.* 2021) and three algorithms implemented within our framework: the Graph Traversal with our quantile search algorithm (GT-q), the algorithm on a 2D conformational landscape (2D MEP), and our energy-aware searching approach. The algorithms were evaluated on two synthetic datasets and the real-world EMPIAR-10076 dataset (Davis *et al.* 2016) with published labels, aiming to identify intermediate transition states and generate trajectories that resemble the ground truth pathway. To reflect realistic conditions where the degrees of freedom are unknown, all models were trained with a fixed dimension (8) for the latent space across all datasets. For more detailed information on data preparation and an introduction to the compared algorithms, please refer to Supplementary Appendix F and B, respectively.

### 3.1 CLEAPA can identify the main transition state and the preferred trajectory in high-dimensional space

**Figure 2.**
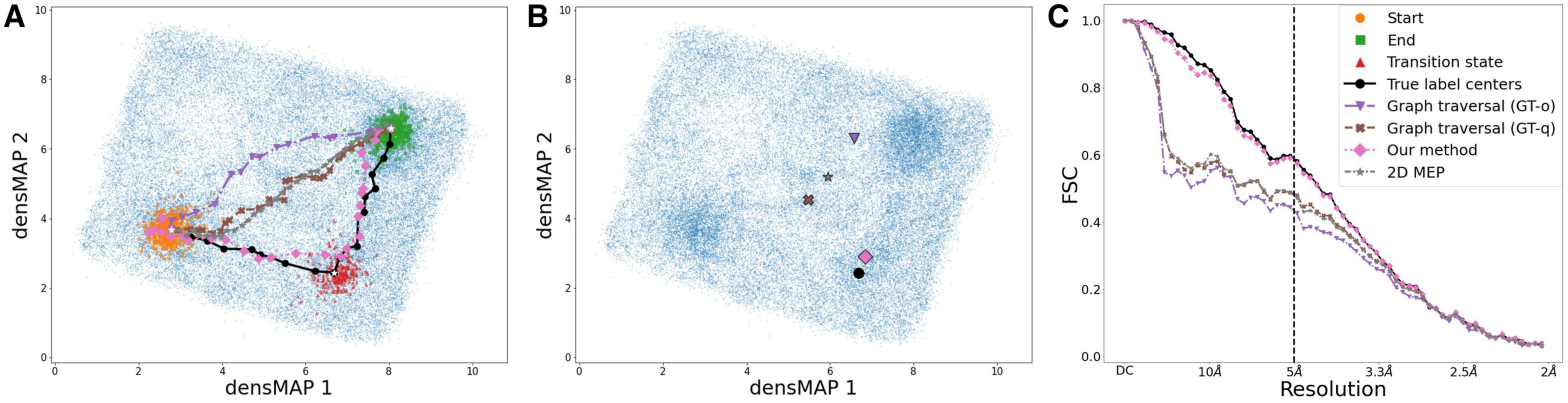
Results of the Hsp90 experiment. (**A**): Paths in the densMAP 2D space are delineated using distinct markers and line styles. True label centers are shown with a solid line and circle markers; GT-o with a dash-dot line and inverted triangle markers; GT-q with a dashed line and ‘X’ markers; our energy-aware method with a dotted line and diamond markers; and 2D MEP with a dashed line and star markers. Start, end, and transition states are indicated by circle, square, and triangle markers, respectively. (**B**): Visualization highlights each path’s closest point to the ground truth transition state, marked by specific symbols. (**C**): Fourier Shell Correlation (FSC) curves illustrate the similarity of each algorithm’s 3D volume to the transition state, utilizing the same markers and line styles as in panel A.

As a proof-of-concept, we applied our methodologies to the Hsp90 dataset (Ali *et al.* 2006), which exhibits two degrees of freedom in conformational changes (Supplementary Figs 16 and 17). Here, only the starting and ending states are given to the algorithms. The performance of the four pathfinding algorithms is depicted in Fig. 2. We assessed each algorithm’s efficacy by its precision in identifying the transition state and accurately pinpointing the preferred trajectory. Initially, we computed the $FSC_{5Å}$ (Equation 6) between the volume generated by the nearest point on the predicted path and the actual 3D volume of the transition state used during the simulation. For reference, the volume derived from the center of the ground truth label for the transition state in the latent space achieved an $FSC_{5Å}$ of 0.582 in the Fourier domain. Our energy-aware method was closely followed, achieving a value of 0.578, as demonstrated in Table 1 and Fig. 2C. In contrast, the GT-q registered a value of 0.480. The 2D MEP method applied to the 2D conformational landscape recorded a value of 0.472, while the GT-o method exhibited the lowest performance, with a value of 0.427. Moreover, the $d_{\text{Transition}}$ metric (Equation 3) demonstrates that our predicted path was closest to the target transition state in the latent space (Fig. 2B).

**Table 1.** Metrics across all datasets.^a^

Hsp90	GT-o	GT-q	2D MEP	Our energy-aware method
$FSC_{5Å}(g_{\mathrm{Transition}},P)$	0.427	0.480	0.472	**0.578**
$d_{\text{Transition}}(g_{\mathrm{Transition}},P)$	3.851	2.907	2.991	**0.897**
$D_{\text{ave}}(G,P)$	2.478	1.789	1.957	**0.628**
$D_{\text{worst}}(G,P)$	3.851	2.907	3.194	**1.214**
**NLRP3**	**GT-o**	**GT-q**	**2D MEP**	**Our method**
$FSC_{5Å}(g_{\mathrm{Transition}},P)$	0.254	0.283	0.403	**0.485**
$d_{\text{Transition}}(g_{\mathrm{Transition}},P)$	3.689	2.915	3.265	**0.602**
$D_{\text{ave}}(G,P)$	2.052	1.477	2.636	**0.587**
$D_{\text{worst}}(G,P)$	3.689	2.915	6.696	**1.154**
**EMPIAR-10076**	**GT-o**	**GT-q**	**2D MEP**	**Our method**
$FSC_{5Å}(g_{C2},P)$	0.887	**0.969**	0.919	0.942
$FSC_{5Å}(g_{E2},P)$	0.905	0.907	0.925	**0.993**
$FSC_{5Å}(g_{E4},P)$	0.923	0.913	0.807	**0.989**
$d_{\text{Transition}}(g_{C2},P)$	2.989	**1.473**	2.759	1.915
$d_{\text{Transition}}(g_{E2},P)$	2.835	2.967	2.177	**0.769**
$d_{\text{Transition}}(g_{E4},P)$	2.710	2.661	3.107	**1.107**
$D_{\text{ave}}(G,P)$	2.936	2.502	3.922	**1.162**
$D_{\text{worst}}(G,P)$	3.706	3.487	8.217	**3.214**

a The algorithms compared GT-o, GT-q, 2D MEP, and our pathfinding algorithm. *g_i_* signifies the state’s center *i* in the latent space, while *P* and *G* denote the sequence of points on the predicted path and the centers of the state on the ground truth path, respectively. Bold values indicate the best metric value across the compared algorithms.

To quantify the overall similarity between the predicted path and the ground truth path, we calculate the average distances between the ground truth path and the predicted path using *D_ave_* (Equation 4). This yields a value of 0.628 for our energy-aware method. Furthermore, the largest discrepancy *D_worst_* (Equation 5) between the trajectories for our method and the ground truth is 1.214, considerably lower than the other methods.

In summary, the findings from our study indicate that our proposed energy-aware method significantly outperforms the others in accurately capturing the transition state and identifying the correct trajectory. The metrics are consistent with the densMAP visualization, further corroborating our results. Both the visualization in Fig. 2A and the calculated metrics clearly demonstrate that our path aligns most closely with the ground truth, significantly outperforming GT-o and 2D MEP in this context. Notably, from the visualization, it is evident that our method is the only one that correctly identifies the transition state.

### 3.2 CLEAPA can identify transition path for molecules with complex dynamics

**Figure 3.**
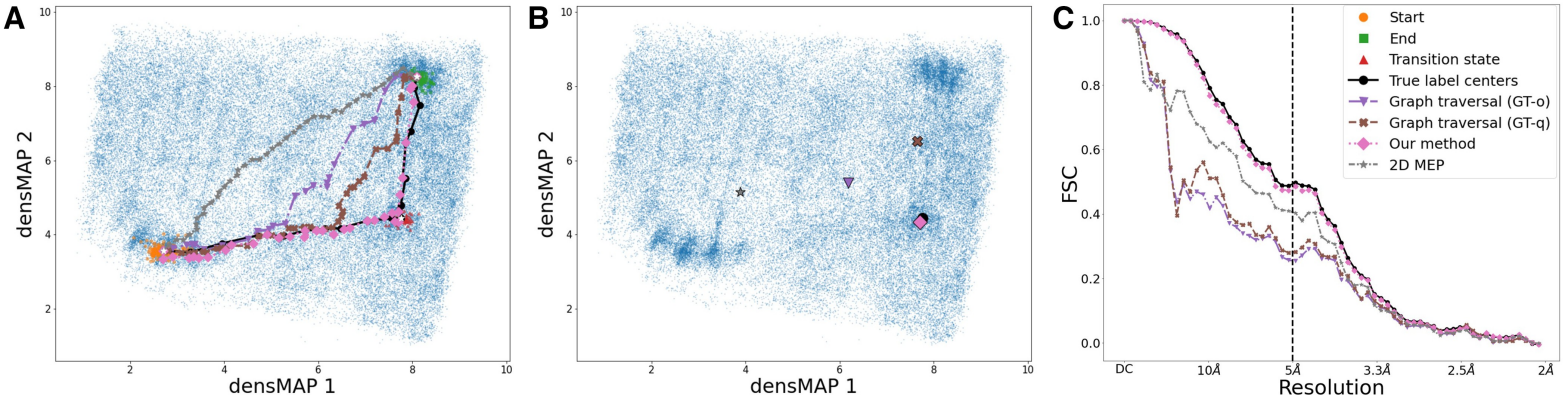
Results of the NLRP3 experiment. (**A**): Paths in densMAP 2D space are differentiated by line styles and markers: True label centers (solid line, circle markers), GT-o (dashdot line, inverted triangle markers), GT-q (dashed line, ‘X’ markers), our energy-aware method (dotted line, diamond markers), and 2D MEP (dashed line, star markers). Start, end, and transition states are denoted by circle, square, and triangle markers, respectively. (**B**): Each path’s nearest point to the ground truth transition state is marked. (**C**): FSC curves compare each algorithm’s volume with the ground truth, using consistent markers and lines for identification.

We then examine the NLRP3 dataset (Sharif *et al.* 2019), wherein the underlying molecule exhibits more complex dynamics, and the conformational motions cannot be captured in two-dimensional space (Supplementary Figs 18 and 19). Four pathfinding algorithms were applied to the recovered conformational landscape with aims similar to the Hsp90 experiment. In the experiment (Fig. 3), the 3D volume from the center of the transition state achieves an $FSC_{5Å}$ of 0.497. Our method demonstrates a comparable correlation with a value of 0.485, as detailed in Table 1. The 2D MEP method achieves a value of 0.403, while the GT-q approach and GT-o method yield values of 0.283 and 0.254, respectively (Fig. 3C). Furthermore, the $d_{\text{Transition}}$ metric indicates that our predicted path is the closest to the target ground truth transition state.

On the other hand, the path located by our method consistently remains closest to the ground truth path. The $D_{\text{ave}}$ metric reveals an average distance of 0.587, and the largest discrepancy is 1.154 according to $D_{\text{worst}}$ (Table 1). The GT-q method ranks second in performance, followed by the GT-o algorithm. In contrast, the 2D MEP approach exhibits a notably lower performance with a $D_{\text{ave}}$ of 2.636 and $D_{\text{worst}}$ of 6.696. Consistent with the visual representation in Fig. 3A and the distance metrics in Table 1, these results from the more complex NLRP3 dataset corroborate the findings from the Hsp90 experiment.

### 3.3 CLEAPA automatically reveals the intermediate states and the transition path in real datasets

**Figure 4.**
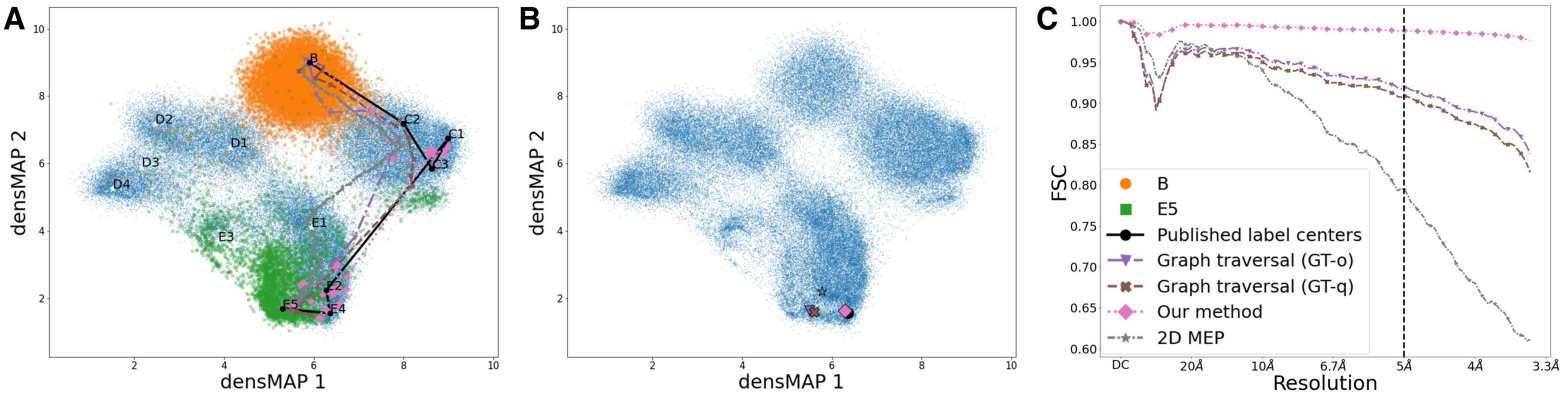
Results of the EMP IAR-10076 dataset. (**A**): Paths in densMAP 2D space are differentiated by line styles and markers: Published label centers (solid line, circle markers), GT-o (dash-dot line, inverted triangle markers), GT-q (dashed line, ‘X’ markers), our method (dotted line, diamond markers), and 2D MEP (dashed line, star markers). States B and E5 are marked with circle and square markers, respectively. (**B**): Shows each path’s closest point to the median in state E4, marked distinctly. (**C**): FSC curves demonstrate comparisons of each algorithm’s volume with state E4, using consistent markers and line styles.

Next, we assessed the four pathfinding algorithms’ performance on the EMPIAR-10076 dataset, renowned for its intricate heterogeneity. Our study specifically targets conformational heterogeneity. Notably, published labels indicate three potential conformational changes occurring from the starting state B to the ending state E5 (Supplementary Fig. 20). In our experiment, we provided only the starting state B and ending state E5 as inputs to each algorithm. The minor classes, previously identified through several rounds of classification, serve as the target transition states that each algorithm aims to discover. The results using the four distinct pathfinding algorithms are depicted in Fig. 4. We utilized the metric $D_{\text{ave}}$ to determine the closeness of the published path and the path identified by each algorithm. According to this metric, both the GT-o and the 2D MEP methods are closest to the second published path, denoted by the orange line in Supplementary Fig. 20, while the other two algorithms are closer to the third published path, represented by the blue line. Notably, both published paths involve transitions through states C2, E2, and E4, allowing these states to serve as benchmarks in assessing the performance. We then generated reference 3D volumes using the median points of these minor states with published labels. Our method achieved the highest $FSC_{5Å}$ values of 0.993 and 0.989 for E2 and E4 states, respectively, as shown in Fig. 4C and Table 1. Similarly, it recorded the smallest $d_{\text{Transition}}$ values at these states.

To further examine the differences between each pathfinding algorithm, we compared their proximity to the published trajectories. For a fair comparison, the path result from GT-o and 2D MEP is compared to the second published path, while other methods are compared to the third published path. Our method achieved the lowest distance scores according to *D_ave_* and *D_worst_*, outperforming the other methods. Notably, except for our algorithm, paths determined by other algorithms lie between two potential published paths, potentially causing ambiguous dynamic movies. Crucially, our energy-aware pathfinding algorithm accurately reveals the correct sequence order in the 2D embedding: in the right-hand region, the order is C2, C3, and C1, and in the bottom region, the order is E2, E4, and E5. This suggests our algorithm not only identifies the target intermediate states but also discerns the correct transition sequence, effectively rejecting unfavorable transitions.

## 4 Discussion and conclusion

Conformational analysis and pathfinding are critical in studying protein function using cryo-EM. In this study, we developed a framework to validate the veracity of paths identified by current computational tools, examining transition paths on the conformational landscape using novel metrics. We created a simulation process capable of emulating continuous conformational motions to establish a ground truth. However, our findings indicate that even with relatively simple synthetic data, current algorithms struggle to accurately identify the ground truth trajectories. To address this limitation, we developed an energy-aware pathfinding algorithm that utilizes energy within the high-dimensional latent space to construct the most probable transition path among heterogeneous biological macromolecules. This approach generates a more plausible path as it takes energy into account. Moreover, the parameters have intuitive meanings, and the algorithm can be used with the densMAP visualization tool for further validation and debugging (Supplementary Appendix D and J).

In terms of implementation, our energy-aware pathfinding algorithm has demonstrated efficient execution times, completing searches within minutes (see Supplementary Appendix K). On the contrary, the performance of the 2D MEP method is notably sensitive to the results of the embedding (see Supplementary Fig. 3). Additionally, optimizing parameters for densMAP introduces extra complexity and extends the time required to construct the 2D landscape in the 2D MEP method. Finally, our algorithm has shown robustness; it has been tested on alternative latent representations learned from EMPIAR 10076, a smaller subset of the Hsp90 dataset and the latent space from 3DVA Punjani and Fleet (2021), consistently identifying similar and correct paths (see Supplementary Appendix G–I). However, our approach has limitations that warrant future exploration. The primary limitation is the assumption about the representation of structural heterogeneity within the latent encoding. Although cryoDRGN is a robust tool for structural analysis, the latent space may not fully capture all heterogeneity aspects. Nonetheless, cryoDRGN’s latent space has proven effective in various real-world analyses of structural heterogeneity, making it a reasonable approximation of the true distribution of biomolecular structures. Another limitation is using Dijkstra’s Algorithm to identify the MEP. In scenarios with many zero-energy edges, our method may struggle, leading to paths through zero-edge-weight regions that may not reflect the most probable or energetically favorable path. To address this, we propose a variant incorporating a kernel function to enhance energy disparities in these regions, potentially identifying a competing MEP. For more details, please refer to Supplementary Appendix J.

Pathfinding across the conformational landscape is an emerging research area that requires further exploration. Developing additional validation tools and pathfinding algorithms can transform these results into a valuable resource for biologists, allowing them to infer protein functions with less effort. To support and stimulate further research, we have consolidated our framework into a modular package, CLEAPA.

## Supplementary Material

btae345_Supplementary_Data

## Data Availability

The dataset, trained cryoDRGN models, 2D embeddings, resulting paths, and other experiment files have been deposited on Zenodo at https://zenodo.org/record/8229902.
