# Supplementary material for: CLEAPA: a framework for exploring the conformational landscape of cryo-EM using energy-aware pathfinding algorithm
Source: Bioinformatics. 2024 Jun 5;40(6):btae345. doi: 10.1093/bioinformatics/btae345 (PMC11167209; doi:10.1093/bioinformatics/btae345)
Supplement: btae345_Supplementary_Data [file btae345_supplementary_data.pdf]

A. Background

A.1. Neural network-based reconstruction model

To recover the 3D density map, state-of-the-art homogeneous reconstruction algorithms use the Expectation-Maximization method to find the maximum a-posteriori probability estimate of the 3D volume V, while marginalizing over the posterior distribution of the unknown viewing angles $\phi = (R, t)$ Scheres (2012b) and other latent variables, as expressed by:

$$V^{MAP} = \underset{V}{\text{argmax}} \sum_{i=1}^{n} \log \int p(I_i|\phi, V)p(\phi)d\phi + \log p(V) \tag{S.1}$$

In practice, this method requires the generation of a low-resolution 3D initial model V^0 based on 2D class averages, common lines in Fourier space, or the stochastic gradient descent algorithm applied to Equation (S.1), and refining this model iteratively to achieve higher resolution.

On the other hand, the heterogeneity reconstruction problem is widely recognized as a primary challenge in single-particle cryo-EM. The standard approach to overcoming this challenge involves reconstructing K discrete volumes and generating $V_1, ..., V_K$ with assignment probabilities π_j, following a similar approach to the homogeneous problem (Equation (S.1)):

$$V_1, ..., V_K = \underset{V_1,...,V_K}{\arg\max} \sum_{i=1}^{n} \log \sum_{j=1}^{K} \pi_j \left(\int p(I_i|\phi, V)p(\phi)d\phi \right) + \sum_{j=1}^{K} \log p(V_j)$$

In practice, heterogeneity issues are often addressed through a hierarchical 3D classification process that involves refining subsets of the imaging dataset. The number of classes and initial models for refinement are manually selected. However, this method struggles when the structural heterogeneity comprises continuous conformational changes Punjani and Fleet (2021a).

CryoDRGN (Deep Reconstructing Generative Networks) Zhong et al. (2021) is one of the first neural network-based approaches for structural heterogeneity reconstruction in single-particle cryo-EM. It utilizes a variational autoencoder (VAE) framework to encode noisy 2D images with unknown viewing angles and imperfect centering into pose-invariant latent variables z that represent the structural heterogeneity. As per the VAE framework, the probabilistic encoder $q_\xi(z|I)$ is a multilayer perceptron with variational parameters ξ. When a 2D cryo-EM image I is inputted, the encoder outputs mean and variance, $\mu_{z|I}$ and $\Sigma_{z|I}$ respectively, statistics that parameterize a normal distribution serving as the variational approximation to the true posterior $p(z|I)$. The latent variable z is priorly set to a standard normal distribution $N(0, \mathbb{I})$. Given latent variable z and Cartesian coordinates $k \in \mathbb{R}^3$ with positional coding, they are passed through the probabilistic decoder $p_\theta(V|k, z)$; the decoder outputs the slice at frequency k in Fourier space. Here, the coordinates are explicitly considered as the positions of each pixel in the 3D Fourier domain, thereby enforcing topological constraints between 2D projections in 3D through the Fourier slice theorem Scheres (2012b):

$$F_2 P_2 = S_2 F_3$$

Here, F_n represents the Fourier transform in n dimensions, P_2 is the projection operator from three to two dimensions, and S_2 is the slice operator, extracting a 2D central slice perpendicular to the projection axis. In essence, each image captures an oriented central slice of the 3D volume in the Fourier domain.

To summarize, for a given input image I_i, the encoder q_ξ generates two variational parameters, $\mu_{z|I_i}$ and $\Sigma_{z|I_i}$. Given the latent variables $z_i \sim N(\mu_{z|I_i}, \Sigma_{z|I_i})$, and the rotation R_i for I_i, the decoder p_θ then reconstructs the image pixel by pixel, taking the positional encoding into account. The microscope's CTF and the phase shift t_i are then applied to the reconstructed pixel intensities. Following the standard VAE framework, the optimization objective function is the variational lower bound of the model evidence:

$$\mathcal{L}(I_i; \xi, \theta) = \mathbb{E}_{q_\xi(z_i|I_i)}[\log p_\theta(I_i|z)] - KL(q_\xi(z|I_i)||p(z))$$

In this scenario, the expectation of the log-likelihood is estimated with a single Monte Carlo sample. This learning process functions as a denoising mechanism, preventing overfitting to noise from a single image, which could lead to increased reconstruction errors for other views. For further details on the rotation estimation, model architecture, and training process, the reader is referred to Zhong et al. (2021, 2019). In addition to producing high-resolution reconstruction results in real-world datasets, the latent variable effectively uncovers structural heterogeneity, paving the way for downstream analyses of structural dynamic processes based on the results from this powerful reconstruction model.

A.2. Energy landscape

The energy landscape is a powerful tool for analyzing structural dynamics. This method is aligned with the fundamental postulate of statistical thermodynamics, which stipulates that in a thermodynamic system of fixed volume, composition, and temperature, all microstates with equivalent energy have equal probabilities Raff (2001). Considering two microstates with corresponding energies E_1 and E_2. As the probabilities for these two microstates depend solely on their energies, we can denote $P_1 = f(E_1)$ and $P_2 = g(E_2)$, where f and g are functions of the energy whose forms are yet to be determined. Now, consider a third microstate which is a composite of microstates 1 and 2. The energy of this third microstate, denoted as E_3, is the sum of E_1 and E_2. The probability of microstate 3 is yet another unknown function h of the energy: $P_3 = h(E_3) = h(E_1 + E_2)$. Since the combined probability of simultaneous observation of

microstates 1 and 2 equals the product of the probabilities of these two microstates, we can write:

$$P_3 = h(E_1 + E_2) = f(E_1)g(E_2)$$

Taking the partial derivatives with respect to E_1 and E_2 on both sides, we obtain:

$$\frac{\partial h(E_3)}{\partial E_1} = g(E_2)\left(\frac{\partial f(E_1)}{E_1}\right) = \left(\frac{\partial h(E_3)}{\partial E_3}\right)\left(\frac{\partial E_3}{\partial E_1}\right) = \left(\frac{\partial h(E_3)}{\partial E_3}\right)$$

and

$$\frac{\partial h(E_3)}{\partial E_2} = f(E_1)\left(\frac{\partial g(E_2)}{E_2}\right) = \left(\frac{\partial h(E_3)}{\partial E_3}\right)\left(\frac{\partial E_3}{\partial E_2}\right) = \left(\frac{\partial h(E_3)}{\partial E_3}\right)$$

Since the right-hand side of both partial derivatives equals $\partial h(E_3)/\partial E_3$, they must be identical. Furthermore, for the derivatives to be equal for distinct values E_1 and E_2, both sides must be constant. We denote this constant as $-\beta$:

$$\frac{\left(\frac{\partial f(E_1)}{\partial E_1}\right)}{f(E_1)} = \frac{\left(\frac{\partial g(E_2)}{\partial E_2}\right)}{g(E_2)} = -\beta$$

After solving these two differential equations, we find:

$$f(E_1) = c\exp(-\beta E_1),\ \ g(E_2) = b\exp(-\beta E_2)$$

Here, c and b are constants. Generally, the probability of the i-th state in a system can be represented as:

$$P_i = A\exp(-\beta E_i)$$

where A can be determined by ensuring the sum of probabilities of all possible states equals one:

$$\sum_{i=1}^{n} P_i = A\sum_{i=1}^{n}\exp(-\beta E_i) = 1;\ \ A \quad = \frac{1}{\sum_{i=1}^{n}\exp(-\beta E_i)}$$

Finally, β can be deduced by studying a known system and generalizing the problem to $\beta = 1/K_BT$, where K_B is the Boltzmann constant and T is the temperature. However, this aspect falls outside the scope of the current study.

In practice, we typically associate the major conformational state with the highest occupancy with the lowest energy, which is set to zero. Therefore, the ratio of the probability of any major conformational state to the lowest energy conformational state α is:

$$\frac{P_i}{P_\alpha} = \frac{\frac{\exp(-E_i/K_BT)}{\sum_{j=1}^{n}\exp(-E_j/K_BT)}}{\frac{\exp(-0/K_BT)}{\sum_{j=1}^{n}\exp(-E_j/K_BT)}} = \exp(-E_i/K_BT).$$

If the number of images in each conformational state is known, the probability ratio can be estimated by calculating the ratio of the number of images in each state. By taking the logarithm, we can derive the energy estimate $\hat{E}_i$ by Fischer et al. (2010):

$$\frac{n_i}{n_\alpha} = \exp(-\hat{E}_i/K_BT);\ \ \hat{E}_i = -\log(\frac{n_i}{n_\alpha})K_BT. \tag{S.2}$$

where n_i and n_α is the number of images correspond to state i and state α.

However, the number of conformational states in a given system is usually unknown for single-particle cryo-EM data. One potential approach is to devise an experiment that takes into account the known conformational change trajectory with timing information Fischer et al. (2010), effectively forcing the possible states to be observable through the microscope. Another common strategy involves selecting the reaction coordinates and dividing them into a set number of bins, each representing a conformation within that specific reaction coordinate. Usually, the number of reaction coordinates is chosen to be two or three, owing to the landscape's complexity and the challenges of searching for the minimum energy path (MEP) in a higher-dimensional space. The energy landscape, with its extensive hills and valleys, can then be navigated to identify an MEP. This path represents the most probable sequence of conformational transitions.

A.3. Mathmatical details of densMAP

DensMAP Narayan et al. (2021) generates embeddings that retain the density information of each point in the original space by utilizing a density measure called the local radius. This local radius is the average distance to the nearest neighbors of a point x_i, denoted $R_\rho(x_i)$. The local radius is defined as the expectation of the distance function over neighbors x_j with respect to a probability distribution $\rho_{j|i}$:

$$R_\rho(x_i) = \mathbf{E}_{j\sim\rho_{j|i}}[d(x_i, x_j)]$$

In line with the Uniform Manifold Approximation and Projection (UMAP) McInnes et al. (2018) framework, the edge probabilities P_{ij} with points x_i in the original space are given as

$$\tilde{P}_{j|i} = \exp(-(||x_i - x_j|| - \text{dist}_i/\gamma_i)$$
$$P_{ij} = \tilde{P}_{j|i} + \tilde{P}_{i|j} - \tilde{P}_{j|i}\tilde{P}_{i|j}.$$

where γ_i is chosen adaptively and corresponds to the length-scale, and dist_i is the distance from x_i to its nearest neighbor. DensMAP renormalizes the edge probabilities P_{ij} to create a probability distribution for calculating the local radius in the original space, $\rho_{j|i} = P_{ij}/\sum_j P_{ij}$:

$$R_P(x_i) = \sum_j \rho_{j|i}||x_i - x_j||^2 = \sum_j \frac{P_{ij}}{\sum_j P_{ij}}||x_i - x_j||^2 \tag{S.3}$$

Following the same concept, the local radius for embeddings R_Q with points y_i in the embedding space is calculated based on the probability distribution $\rho_{j|i} = Q_{ij}/\sum_j Q_{ij}$:

$$R_Q(y_i) = \sum_j \rho_{j|i}||y_i - y_j||^2 = \sum_j \frac{Q_{ij}}{\sum_j Q_{ij}}||y_i - y_j||^2 \tag{S.4}$$

where Q_{ij} represents the edge probabilities computed in the embedding space:

$$Q_{j|i}(a, b) = (1 + ad_{ij}^{2b})^{-1}$$

where d_{ij} represents the distance between i and j in the embedding, and a and b are additional shape parameters. Finally, densMAP modifies the loss function of UMAP by incorporating the correlation of the logarithms of the local radius in the original space and the embedding space. The densMAP loss function is optimized as follows:

$$\mathcal{L}^{\text{densMAP}} = \text{CE}(P||Q) - \lambda\text{Corr}(\log R_P, \log R_Q)$$

Here, CE represents cross-entropy, and λ is a user-chosen parameter that determines the importance of the density preservation term.

B. The Implementation of other pathfinding algorithms

In contrast to the reconstruction process of 3D density maps, the analysis of structural dynamics in single-particle cryo-EM is still evolving and remains relatively novel. Results from different methods may necessitate entirely distinct analytical approaches. In this section, we outline various methods for identifying the most probable sequence of transition states occurring between any two conformational states. In principle, many conformational paths can connect a start to an end conformation. However, most of these paths involve high-energy states, rarely populated under biologically relevant conditions. Due to the exponential nature of the Boltzmann inverse relationship between energy and occupation probability (Equation (S.2)), the lowest-energy conformational paths contribute maximally to function. Therefore, the search for the preferred path can be reformulated as a search for the MEP path.

As many reconstruction models depict structural heterogeneity as an n-dimensional manifold, the simplest and most intuitive approach involves sampling a pathway along the most variant coordinate. This can be accomplished by first using Principal Component Analysis Penczek et al. (2011a) or its variants Tagare et al. (2015); Punjani and Fleet (2021a) to embed the heterogeneity representation coding, followed by sampling along the first or second component known as eigen-analysis. However, due to the complexity of biomolecular dynamics, this approach might not provide the most probable pathway. Moreover, sampling in only one or two directions may be insufficient to capture the true underlying conformational transitions.

The cryoDRGN team proposed an innovative method, known as graph traversal Zhong et al. (2021); Zhong (2022), for exploring trajectories within the latent space. This algorithm commences by creating a nearest neighbor graph from the latent encodings. A neighbor is defined if the Euclidean distance is below a threshold, computed from the statistics of all pairwise distances [1]. The edges are subsequently pruned to ensure distant neighbors are disconnected from the given vertex. The Dijkstra algorithm Cormen et al. (2022) is then deployed to find the shortest path connecting a series of points along the graph. The method selects the median of all pairwise distances in the graph as the pruning point. Consequently, the resulting trajectory is less likely to pass through a low-occupancy region as distant neighbors are disconnected. This method can uncover more complex transition paths by searching in a higher-dimensional space. However, since the edge weights of the graph are defined as Euclidean distances, it may obtain suboptimal results when exploring conformational changes in biomolecules. Additionally, it is evident that the threshold selection impacts the final generated trajectories. To address this, we implement a variant of the original method that performs a hyperparameter search on the threshold distance using quantiles in this work. The best hyperparameter is chosen based on the procedure described in Appendix D. In the comparison of different pathfinding algorithms, we refer to the original version of graph traversal as GT-o and our variant as GT-q.

Another set of approaches involves searching for the MEP within an energy landscape. The first approach is grounded in graph theory, where a graph is constructed and the most optimal path is identified based on a specified metric, such as the shortest path on the graph Marcos-Alcalde et al. (2015). This method can be adapted to higher-dimensional energy landscapes Marcos-Alcalde et al. (2020); however, constructing a high-dimensional energy landscape for cryo-EM datasets currently presents a challenge due to the lack of efficient methods, to the best of our knowledge. The second approach incorporates the string method Wu et al. (2022b); Weinan et al. (2002),

[1] In graph traversal, `max_neighbors` refers to the number of neighbors utilized in constructing the nearest neighborhood graph. Meanwhile, `avg_neighbors` determines the threshold for graph pruning, with a range of `(1, max_neighbors)`. The default parameters are set as `max_neighbor = 10` and `avg_neighbors = 5`.

which first connects two predetermined states with a straight line. This path is subsequently updated in a direction perpendicular to the gradient of the energy surface, continuing until the difference between successive updates falls beneath a user-determined tolerance. Nevertheless, this method may be affected by the selection of initial states and the determination of the tolerance value. Furthermore, its performance substantially degrades when searching within complex energy landscapes.

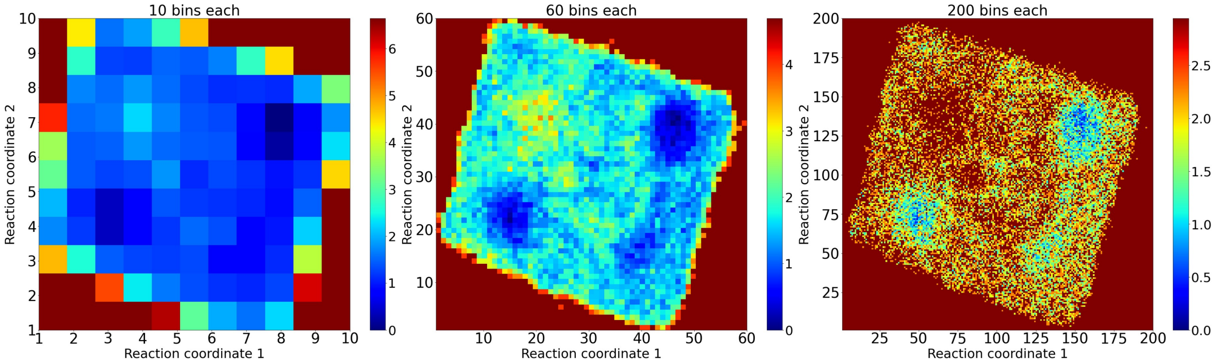

Supplementary Fig. 1: The impact of varying numbers of bins for constructing a 2D conformational landscape, with the number of bins increasing from left to right. The color represents the energy of each grid cell. As the number of bins increases, many grid cells reach the maximum energy, resulting in a more distinct differentiation of energy levels within the landscape. Conversely, when a smaller number of bins is used, more grid cells exhibit zero energy, leading to a flatter landscape with fewer valleys.

Lastly, the POLARIS (Path of Least Action Recursive Survey) algorithm seeks the MEP in a 2D energy landscape using a recursive method Seitz and Frank (2020). It divides the landscape into equal 4^n blocks, determined by a user-defined parameter n, and pinpoints the minimum energy points in each block as candidates for the searching process. Another user-defined parameter, r, specifies the number of candidate transition points included in the search process. The search process recursively subdivides the potential path into pairs of points and searches based on these pairs with the same number of r until subdivision no longer reduces the energy. For instance, if the user-defined parameters are $n = 1$ and $r = 1$ with a starting point S and an ending point E, the possible path would involve searching from $S \to C_i \to E$ for four candidate transition points, where $i = 1, \dots, 4$. Subsequently, for the path $S \to C_1$, the algorithm would look for any other possible candidates to minimize the energy, thus refining the path further. This method allows a more thorough exploration of the energy landscape, as every point in the landscape can be a candidate when n is set to the maximum. Moreover, setting the parameter $r = 1$ can substantially reduce computation time, rendering the POLARIS method a promising option for searching MEPs in 2D energy landscapes. However, computation time would increase dramatically when applying similar searching processes to higher-dimensional energy landscapes. Thus, this approach is extremely sensitive to the choice of a dimension reduction method capable of projecting higher-dimensional encodings to 2D and constructing an accurate 2D energy landscape. In this study, we incorporate POLARIS to evaluate its efficiency in navigating the 2D conformational landscape in conjunction with recent neural network-based reconstruction models. The integration process includes performing dimension reduction and building a 2D histogram on the embedding space as described in Algorithm 2.

In this study, The 2D conformational landscape was constructed with 60 bins for each coordinate for the synthetic dataset and 100 bins for the EMPIAR-10076 dataset. In addition, the transition state weighting of POLARIS is turned on to avoid traversing over high-energy regions.

Algorithm 2 Proposed 2D MEP Algorithm

Require: Latent space z, two points A and B in the latent space, number of bins b, parameters of densMAP and POLARIS.
Ensure: $2D\_MEP$
1: Embed z into a 2D embedding using densMAP.
2: Divide the 2D embedding space into b bins to create a grid. Count the points within each grid cell to estimate their occupancies, n_b (Supplementary Fig. 1).
3: Construct a 2D conformational landscape using the occupancies n_b and the Boltzmann relation, as shown in Equation (S.2).
4: Utilize the POLARIS algorithm to identify the $2D\_MEP$ within the 2D conformational landscape.

C. Examining the impact of 2D embedding on POLARIS

In this section, we discuss the details and constraints of searching on a 2D conformational landscape using POLARIS. As noted in the main text, we opted for a density-preserving manifold embedding technique over commonly used approaches such as UMAP or

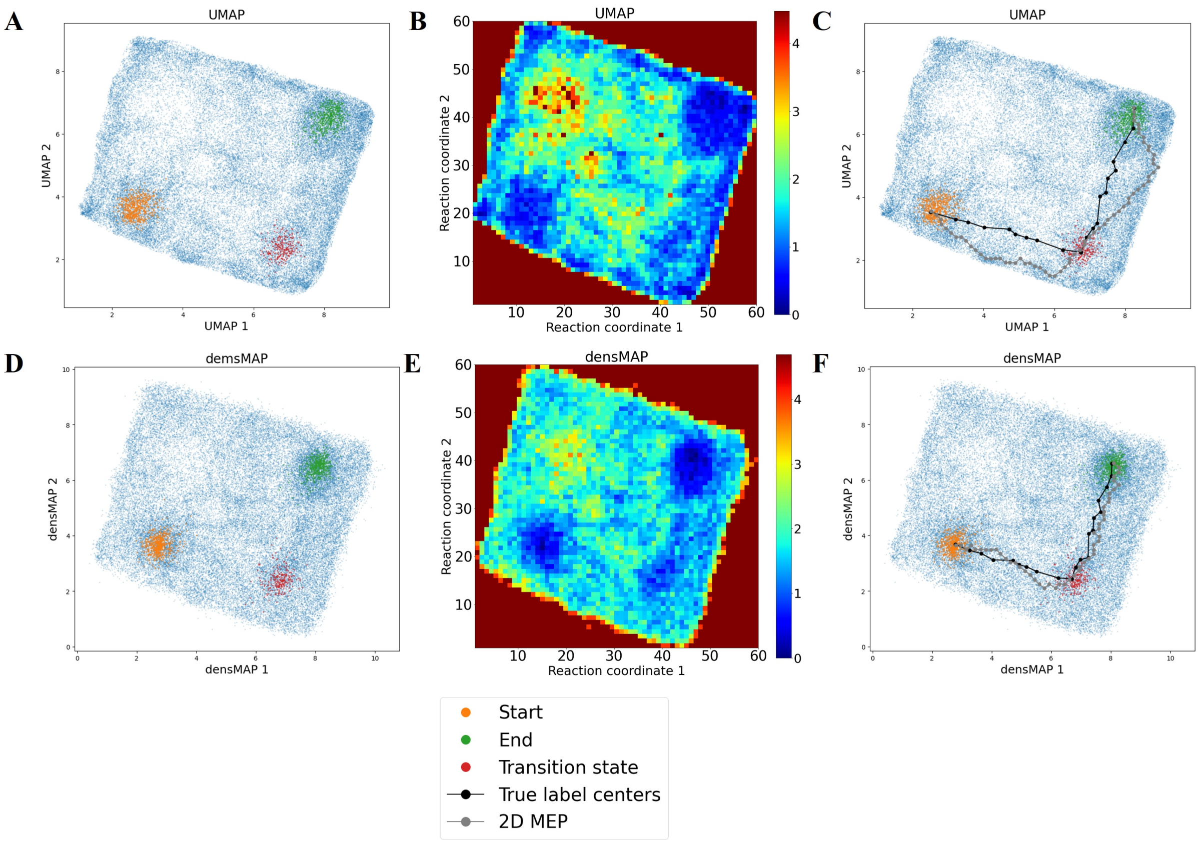

Supplementary Fig. 2: Comparison of the impact of embedding selection when using POLARIS to search for the MEP on a 2D conformational landscape. **A** displays a 2D visualization after performing UMAP on the Hsp90 experiment latent space. Orange dots represent the starting state, while green dots denote the ending state. Red dots signify the transition state. **B** shows the 2D conformational landscape constructed by a 2D histogram after selecting 60 bins for each coordinate, and **C** contrasts the path identified by POLARIS (depicted by the gray line) with the ground truth transition sequence (represented by the black line). Figures **D**, **E**, and **F** represent results obtained using densMAP, corresponding to the same types of visualizations as in **A**, **B**, and **C**. In the UMAP scenario, the low-energy region is shifted towards the landscape's edge, which inaccurately represents the transition sequence's center. Conversely, when employing densMAP, the low-energy regions accurately align with the ground truth center sequence. Note that in this experiment, the transition state center is also provided to the algorithm in addition to the start and end state.

t-Distributed Stochastic Neighbor Embedding (t-SNE) Van der Maaten and Hinton (2008). This choice is critical when using the bin-counts approach to construct a 2D conformational landscape (Appendix A.2). To demonstrate its significance, we compared embeddings using UMAP and densMAP in the Hsp90 experiment (Supplementary Fig. 2). After defining the starting, ending, and transition state centers as inputs, our objective was to identify a path akin to the black line, symbolizing the actual center of the transition sequence. Empirically, we found that the low-energy (high-density) region using UMAP differs from the one using densMAP and may distort the 2D conformational landscape. This difference influences the result of minimum energy pathfinding by POLARIS, leading to the predicted pathway deviating from the true sequence.

The other factor that may impact the final selected path is the relative position of the start, end, and transition center on the 2D embedding. To illustrate this impact, we used the same occupancy map to create a new dataset in which the first conformational change is reduced to 0.5 degrees for each state, and the second conformational change is reduced to 1.44 degrees for each state. This new dataset was created following the same workflow as in the Hsp90 experiment, and we used the same training processes to obtain the latent space. Subsequently, we constructed a 2D conformational landscape using the same bin size and applied the same parameters in POLARIS to find the MEP. The results are presented in Supplementary Fig. 3.

When comparing with the true sequence, we observed that even though the 2D conformational landscape looks similar (Supplementary Fig. 3A and Supplementary Fig. 3C), with three regions corresponding to the starting, ending, and transition states, the outcomes are completely different. The new dataset accurately reveals the path. This difference can be attributed to the fact that, in these cases, the regions within the start, end, and transition areas have roughly the same energy. Consequently, if no significant low-energy regions are present to explore, the MEP would depend more on the length of the distance between these states. In the dataset with lesser rotation and movement, the distance between the starting and ending points is 25.239, which is larger than the original dataset's 24.352, and

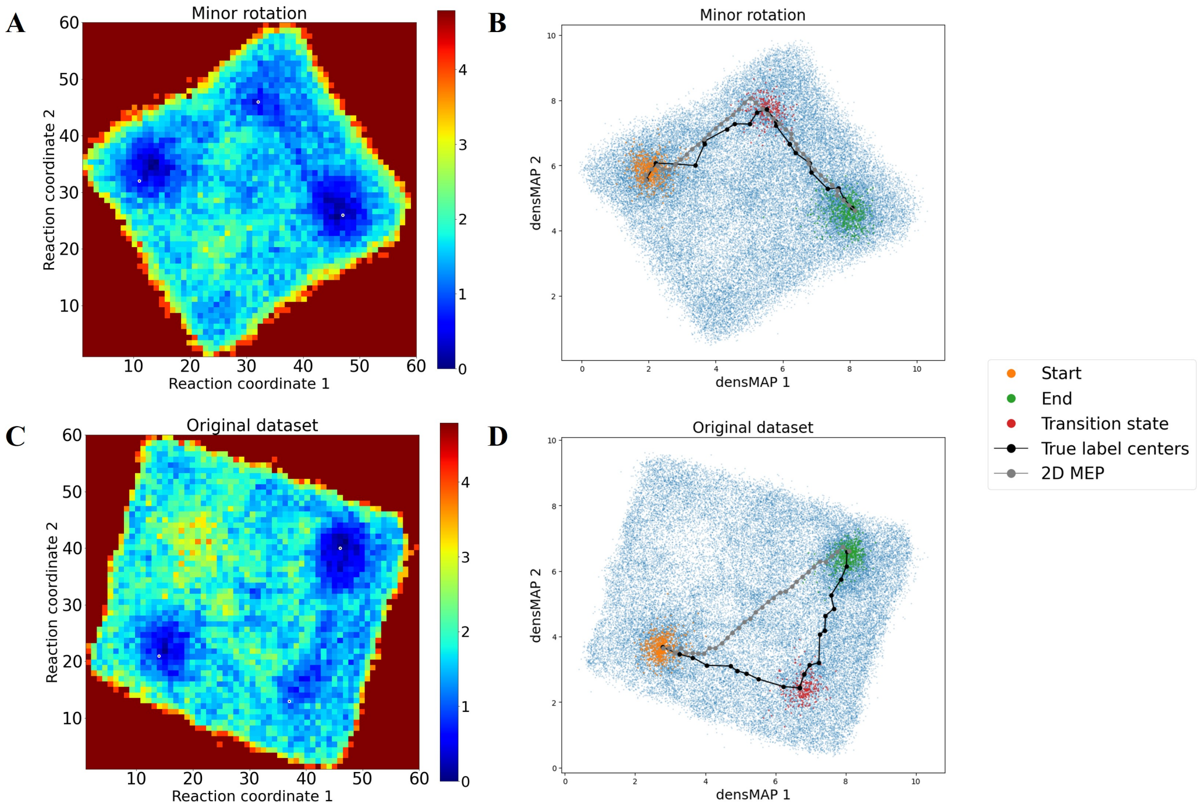

Supplementary Fig. 3: Comparison of the new dataset, with less rotation and movement, when searching for the MEP using POLARIS. **A** and **B** represent results from the new dataset, in which the first conformational changes are reduced to 0.5 degrees each, and the second conformational changes are reduced to 1.44 degrees each for the Hsp90 molecule. **A** shows the 2D conformational landscape constructed after embedding using densMAP, with 60 bins selected for each coordinate. **B** contrasts the predicted path (depicted by the gray line) with the ground truth center sequence (represented by the black line). **C** and **D** depict the results from the original Hsp90 experiment, using the same representations as in **A** and **B**. Although the two conformational landscapes appear highly similar, the paths found by POLARIS are entirely different. The predictions on the dataset with less rotation and movement accurately capture the transition state region.

the distance passing through the transition state is 61.497 smaller than the original dataset's 65.676. Therefore, POLARIS explores the transition state region instead of traversing over the middle region for the dataset with lesser rotation and movement. These effects substantially impact performance and introduce uncertainty when using POLARIS to search on a 2D conformational landscape.

D. Rationale Behind Our Local Density Estimation and the Proposed Quantile Searching Algorithm

The local density estimation for n_v, as described in the main text of our energy-aware pathfinding algorithm, offers several advantages. Firstly, it intuitively establishes a topological-like grid akin to 2D grids in function. Secondly, it is computationally more efficient than alternative methods such as the local radius used in densMAP (Equation (S.3)) or kernel density estimation. Thirdly, this method demonstrates robustness against noisy data points, as the threshold θ serves as a rejection boundary to exclude outliers.

Furthermore, our algorithm can be paired with visualization techniques to provide users with deeper insights into the dataset. The selection of θ influences the overall morphology and smoothness of the conformational landscape. For example, a smaller θ leads to fewer neighbors remaining within the threshold for many nodes, creating a landscape characterized by numerous hills and fewer valleys. In contrast, a larger θ results in more neighbors staying within the threshold, producing a flatter landscape (Supplementary Fig. 4). This effect can be likened to constructing a 2D conformational landscape with a variable number of bins along each coordinate (Supplementary Fig. 1), where a greater number of grid cells equates to a smaller threshold for graph pruning. This scenario typically results in a higher incidence of zero counts within the grids (indicating higher energy), thereby accentuating the energy differences within the landscape, marked by more distinctive hills and valleys.

In our study, we found that employing quantiles of pairwise distances between nodes throughout the graph facilitates a systematic method for selecting θ to estimate n_v. An optimal threshold can be determined by keeping the zero energy ratio of the entire landscape within a predefined range (r_l, r_u). Empirically, a range of $r_l = 0.01$ to $r_u = 0.1$ has been effective in pinpointing the optimal quantile for the threshold. For the purpose of threshold searching, we typically use quantiles from 0.1 to 0.9, with increments of 0.1. It is noteworthy

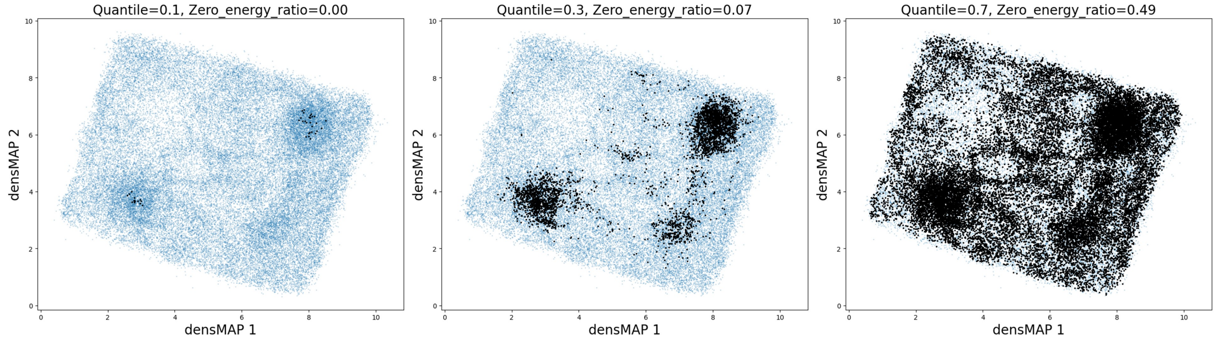

Supplementary Fig. 4: 2D visualization of the graph using various quantiles as thresholds in our energy-aware pathfinding algorithm, with threshold values increasing from left to right. The black points denote zero-energy points. As the threshold (expressed in terms of quantiles) increases, the number of zero-energy points within the landscape also increases. Notably, the pattern of these zero-energy points aligns closely with the low-energy region illustrated in Supplementary Fig. 1.

that the identified threshold through this method may not be unique. To identify the most appropriate threshold, we calculate the average distance between each node $p_1, ..., p_q$ and their respective neighbors r_{ij} along the path, subsequently averaging these distances for each path:

$$\frac{1}{q}\sum_{i=1}^{q}\frac{\sum_j ||p_i - r_{ij}||}{N} \tag{S.5}$$

The path exhibiting the lowest mean distance is chosen, as nodes on a lower energy path are typically closer to their neighbors. This selection process ensures the identification of the most coherent and energetically favorable path.

E. The cryoDRGN can capture the energy landscape with enough precision

In this section, we evaluate the capability of the current reconstruction model to extract conformational landscapes from cryo-EM images. According to a recent study Dsouza et al. (2023), Manifold-EM Seitz et al. (2022) and cryoDRGN Zhong et al. (2021) are the two most promising methods for recovering the underlying conformational landscape. In this study, we chose cryoDRGN because it requires less hyperparameter tuning and can efficiently extract a conformational landscape with dimensionality larger than two [2]. However, we considered a more realistic setting compared to Dsouza et al. (2023). Firstly, we assumed that the underlying degree of freedom of the conformational landscape is unknown, so we always learned an 8-dimensional latent space in all the experiments and used a density-preserving dimension reduction algorithm densMAP for visual assessment. Additionally, we accounted for the CTF in the forward model and set the SNR to a lower value of 0.1 instead of 1 to make the synthetic dataset more closely resemble a realistic dataset.

We first examined the two synthetic datasets created for this study. The ground truth occupancy map of Hsp90 is designed to have two degrees of freedom, with two major states located in the lower-left and upper-right corners, respectively. These states show a high occupancy level, as demonstrated in Supplementary Fig. 17. A transition state is added in the lower-right corner with higher occupancy than the background but less occupied than the major states. For the NLRP3 dataset, we considered a more complex conformational dynamic than in the Hsp90 experiment. The occupancy map was similarly designed but had three degrees of freedom as illustrated in Supplementary Fig. 19. The starting and ending states, located in the corner of the 3D box, have the highest occupancies. A transition group in another corner exhibits slightly lower occupancy, though it is still higher than the background occupancy. The noise was added to the background to mimic the real-world scenario for both datasets. For the EMPIAR-10076 dataset, we do not have the ground truth occupancy map, but we do have the published labels for each major and minor state as well as the transition path (Supplementary Fig. 20) released by a previous study Davis et al. (2016).

The conformational landscapes of the two synthetic datasets, as recovered by cryoDRGN, can be visualized in Fig. 2 and Fig. 3 in the main text using the densMAP. It's clear that cryoDRGN successfully captures the two major states and transition states at the corner, all of which have a higher occupancy than the background. In addition, the major states enjoy a higher occupancy level than the transition state. For the EMPIAR-10076 dataset, the recovered landscape is illustrated in Supplementary Fig. 21, and the optimized conformational landscape by the analyze-landscape pipeline (Zhong (2022) and Appendix F.4) is depicted in Supplementary Fig. 22. We assigned different colors to data points that belong to different classes according to the label. Clearly, the conformational landscape successfully separates each major and minor state. In addition, states that are closer in the plot of the transitional path (Supplementary Fig. 20) also appear closer on the scatter plot. From these observations, we infer that cryoDRGN can learn a reasonably accurate conformational landscape from both synthetic and real datasets.

[2] At present, the Manifold-EM software requires significant human intervention for hyperparameter tuning and does not support adjusting the dimensionality of the latent space to higher dimensionality.

F. Data preparation

F.1. Data preparation of the Hsp90 dataset

We generate a simulated dataset featuring two degrees of freedom in conformational changes based on the Hsp90 molecule (PDB ID: 2CG9) Ali et al. (2006). The dual arm-like structures of this molecule allow us to conveniently depict two distinct directions of conformational changes. For each of the conformational changes, we create 20 states. The first direction pertains to the movement of one arm by 1 degrees for each state, and the second direction involves rotating the other arm by 2 degrees for each state (Supplementary Fig. 16). These two directions correspond to the two coordinates in the occupancy maps. Using the synthetic process detailed in the previous subsection, we transform these conformational changes into 3D density maps. These maps, derived from atomic models, possess a resolution of 3 Å, a box size of 128, and a pixel size of 1 Å.

Subsequently, we devise an occupancy map with three groups of occupancies to simulate different conformational states of the Hsp90 molecule (Supplementary Fig. 17). Two high-occupancy groups located in the lower-left and upper-right regions of the map, respectively, represent the initial and final states. Another group, with an occupancy that surpasses the background but is lower than the starting and ending states, is situated in the lower-right region of the map. This group signifies the main transition state that we aim to discover. To emulate the transition process and increase the chance of capturing more images during the conformational change, we also include a path connecting these three groups in the occupancy map. For a more realistic simulation, we introduce random noise into the background occupancy rather than using a constant 0 occupancy. This accounts for potential captures of conformations not part of the transition process in the dataset. The inclusion of random noise raises the complexity of the experiment, as pathfinding algorithms now have to differentiate between true transition states and random noise. After generating clones for each conformational state using the occupancy map, we construct 50 random viewing angles and project these clones into particle images. To achieve an SNR of 0.1, these images are convolved with a point spread function, and Gaussian noise is added. The resulting $56,000$ particle images constitute our dataset, which serves as the input for cryoDRGN.

F.2. Data preparation of the NLRP3 dataset

For simulating more intricate conformational changes, we utilize the NLRP3 molecule (PDB ID: 6NPY) Sharif et al. (2019). This molecule, which comprises a head, an arm, and a binding site ball adjacent to the arm, allows us to design three degrees of freedom in conformational change. For each of the conformational changes, we generate 20 states. The first change involves rotating the head by 2 degrees for each state, the second involves moving the binding ball by 2 degrees for each state, and the final one is represented by moving the arm by 2 degrees for each state, as shown in Supplementary Fig. 18. Using the synthetic process, we transform these conformational changes into 3D density maps. These maps, derived from atomic models, possess a resolution of 3 Å, a box size of 128, and a pixel size of 1 Å.

Following the methodology outlined in the simulation process, we create an occupancy map to simulate the transition process in the NLRP3 experiment. The occupancy map is designed similarly to the Hsp90 experiment, where the starting and ending groups have the highest occupancies, and a transition group has lower occupancy but is still higher than the background occupancy. We set 0 for the background occupancy and introduce $4,000$ random points as noise to mimic the real scenario. Additionally, to keep the computational requirements in check, we employ only 20 random viewing angles for generating the 2D images. Subsequently, these images are convolved with a point spread function, and Gaussian noise is added to reach a signal-to-noise ratio of 0.1. The final dataset, constituting 75,560 particle images, is then fed into cryoDRGN for further analysis.

F.3. Data preparation of the EMPIAR-10076 dataset

To concentrate on the identification of potential transition states and simplify the training process, we adhere to the same workflow and use the intermediate results from the cryoDRGN team Kinman et al. (2023). The box size is 256 pixel and the pixel size is 1.64 Å after downsampling. We use the published labels Davis et al. (2016) as our ground truth to avoid reliance on clustering for analyzing structural heterogeneity. We remove states with labels A, F, and Discarded as they do not contribute to the conformational transitions from B to E5 (Supplementary Fig. 21A) so that the remaining number of particles in the dataset is 87,461. Subsequently, we leverage the median points of B and E5 as the starting and ending points for each algorithm. Finally, to calculate the metrics, the reference volume is generated at the center of the transition state using the published label.

F.4. Implementing the analyze-landscape pipeline for the EMPIAR-10076 dataset

We follow the cryoDRGN team's three-step pipeline to map latent encodings into a more interpretable space represented by 3D volumes. The first step involves sketching the latent space. Given the high computational cost of generating a 3D volume for every point in the latent space, we simplify the latent space into a set of representative samples. This is achieved by using the centers of clusters identified by KMeans clustering, with the number of clusters set to 500. Subsequently, we generate 3D volumes with the cryoDRGN decoder using these selected points. The distribution of these representative 3D volumes represents the approximation of the population of the target protein.

The second step involves masking and dimension reduction. We apply masks to these volumes to filter out the background and focus on variations within the volumes. Subsequently, Principal Component Analysis is applied directly to these masked volumes to reduce their dimensionality to a desirable level. The reduced dimensionality is set to 8, equivalent to the original latent space dimension.

The final step involves learning a mapping from the latent space to the newly constructed volume space. To accomplish this, we require more training data points. This is achieved by uniformly selecting additional 1000 points from the latent space. These points are

used to generate additional volumes. We then apply the same mask created in the first step to these volumes and transform these 3D volumes into the same reduced dimensionality. A Multilayer Perceptron network is subsequently trained to learn the mapping between the latent and volume spaces. Once the model is trained, it is used to predict the volume space of the entire dataset. The final volume space is used as the optimized latent space as shown in (Supplementary Fig. 22).

F.5. CryoDRGN training and generation of 3D volumes

For the training process on the two synthetic datasets, we follow the default architecture and training parameters as specified by cryoDRGN, albeit with a modification in the number of nodes in the hidden layers; this is adjusted to 256 to expedite the experimental process. Given that our objective is to focus on comparing the performance of different pathfinding algorithms, we feed the ground truth poses and CTF parameters into the reconstruction model. The generation of 3D volumes is conducted using the decoder of the trained cryoDRGN model. The Fourier Shell Correlation (FSC) is calculated using a custom script that can be accessed in the code repository.

F.6. Experiment details

In this study, we set the learning rate for densMAP to 0.001 across all datasets, with an adjustment to 0.005 for datasets undergoing analyze-landscape (Appendix F.4). Additionally, the parameters `dens_frac` and `dens_lambda` were set to 0.7 and 3.0, respectively, for the two synthetic datasets, while all other parameters remained at their default settings. In the pathfinding experiments, we directly input the starting and ending centers for both the graph traversal algorithm and our energy-aware pathfinding algorithm. To navigate the 2D MEP using POLARIS, we first constructed a 2D conformational landscape from the densMAP 2D embedding space utilizing a 2D histogram. We then input the corresponding starting and ending centers from the embedding space. For the synthetic dataset, we considered the mean of data points belonging to the same state in the latent space as the centers. Conversely, for the real dataset, we opted for the median of the data points to mitigate the influence of outliers.

F.7. Data availability

The synthetic datasets were generated using custom code available at the following GitHub: https://github.com/tengyulin/synth_hsp90 and https://github.com/tengyulin/synth_nlrp3. The original EMPIAR-10076 dataset can be accessed at EMPIAR-10076. The trained cryoDRGN models, 2D embeddings, resulting paths and other experiment files have been deposited on Zenodo at https://zenodo.org/record/8229902.

Table 1. Experimental results for different metrics in the EMPIAR 10076 datasets after analyze-landscape. The algorithms compared are Graph Traversal (GT-o), Graph Traversal with quantile threshold search (GT-q), 2D embedding with POLARIS (2D MEP), and our energy-aware pathfinding algorithm. GT-o was unable to identify a path in this dataset. g_i signifies the state's center i in the latent space, while P and G denote the sequence of points on the predicted path and the center points of the state on the published path, respectively.

EMPIAR-10076 after analyze-landscape	GT-o	GT-q	MEP(2D)	Our method
$FSC_{5\text{Å}}(g_{C2}, P)$	N/A	0.852	0.933	0.894
$FSC_{5\text{Å}}(g_{E2}, P)$	N/A	0.904	0.938	0.962
$FSC_{5\text{Å}}(g_{E4}, P)$	N/A	0.817	0.917	0.932
$d_{\text{Transition}}(g_{C2}, P)$	N/A	3.294	2.132	2.175
$d_{\text{Transition}}(g_{E2}, P)$	N/A	3.388	2.505	1.787
$d_{\text{Transition}}(g_{E4}, P)$	N/A	3.079	2.400	2.318
$D_{\text{ave}}(G, P)$	N/A	2.972	3.884	2.427
$D_{\text{worst}}(G, P)$	N/A	10.569	9.430	8.525

G. The robustness of the framework when dealing with different latent space

Given the inherent complexity of real-world datasets, the latent encodings that are learned might not always sufficiently capture structural heterogeneity. This shortfall can potentially result in suboptimal outcomes during the search phase of the pathfinding algorithm. To mitigate this limitation, we integrate a novel downstream analysis pipeline, termed analyze-landscape, into our framework as a post-processing step. This pipeline, as proposed by Zhong (2022), primarily aims to map the latent space onto a more interpretable conformation space. It achieves this by capturing the heterogeneity in the output space formed by representative 3D volumes. We conducted experiments with this optimization to assess its impact on the pathfinding algorithm. This example can also examine the robustness of the framework when dealing with different latent spaces.

The resulting space is depicted in Supplementary Fig. 5. Notably, the minor states' location on the 2D embedding aligns more coherently with the transition graph shown in Supplementary Fig. 20. In particular, the published trajectory no longer intersects itself in the C and E regions of the 2D space. Subsequently, we conducted a comparable experiment in this refined space, analogous to our approach with the original space, using the centers of states B and E5 as starting and ending points. The results are presented in Supplementary Fig. 5A. In this instance, the GT-o algorithm failed to find a path. Employing D_{ave} as the metric, the GT-q approach identified the second path. Conversely, the other methods highlighted the third path. This outcome justifies the necessity of performing a quantile hyperparameter search for the graph traversal algorithm, as recommended in our framework.

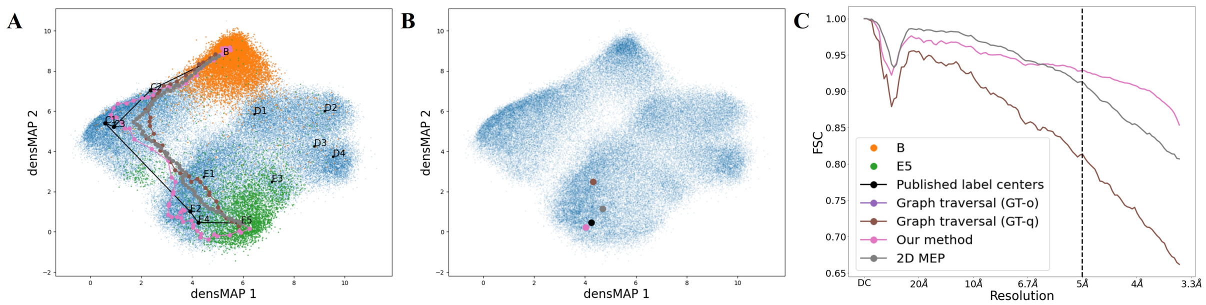

Supplementary Fig. 5: The results of the EMPIAR-10076 dataset after the analyze-landscape pipeline. **A**: Paths from various algorithms in the densMAP 2D space with orange, green, and red dots representing starting, ending, and transition states, respectively. Published trajectory is a black line. Paths are color-coded: GT-o in purple, GT-q in brown, 2D MEP in gray, and our energy-aware method in pink. **B**: Shows the closest point of each path to the published label's median point in state E4 in latent space. **C**: FSC curves comparing each algorithm's volume with the volume in state E4, utilizing the closest volume to the published label's center in state E4 for each path.

For evaluative comparison, the shared states between the second and third paths were C2, E2, and E4. When calculating $FSC_{5\text{Å}}$ relative to the volumes produced by the center of these minor states, our method yielded the top scores of 0.962 and 0.932 for the E2 and E4 states, respectively, as shown in Supplementary Table 1. The $d_{\text{Transition}}$ metric reflects a similar pattern, with the path identified by our methodology being the nearest to the E2 and E4 states. Furthermore, according to the overall distance metric, our method exhibited the closest proximity to the published path, registering an average distance of 2.427 as represented by D_{ave}. Additionally, it achieved a value of 8.525 in terms of D_{worst}, illustrating that our energy-aware pathfinding method consistently outperforms the others in this new space. The results suggest that our proposed method is robust against changes in the underlying conformation space.

Table 2. Experimental results for various metrics in the subset from the Hsp90 dataset, which contains 30,000 images, are presented. In this analysis, we report the mean and standard deviation of the metrics over five independent runs. The algorithms compared include GT-o, GT-q, 2D MEP, and the energy-aware pathfinding algorithm. Here, g_i signifies the center of state i in the latent space, while P and G denote the sequence of points on the predicted path and the center points of the states on the ground truth path, respectively.

Hsp90	GT-o	GT-q	2D MEP	Our energy-aware method
$FSC_{5\text{Å}}(g_{Transition}, P)$	0.441 (0.021)	0.486 (0.042)	0.467 (0.024)	**0.534** (0.007)
$d_{\text{Transition}}(g_{Transition}, P)$	3.389 (0.511)	2.409 (1.413)	2.931 (0.325)	**0.945** (0.333)
$D_{\text{ave}}(G, P)$	2.111 (0.335)	1.476 (0.722)	1.865 (0.204)	**0.660** (0.206)
$D_{\text{worst}}(G, P)$	3.393 (0.507)	2.529 (1.248)	3.119 (0.290)	**1.316** (0.327)

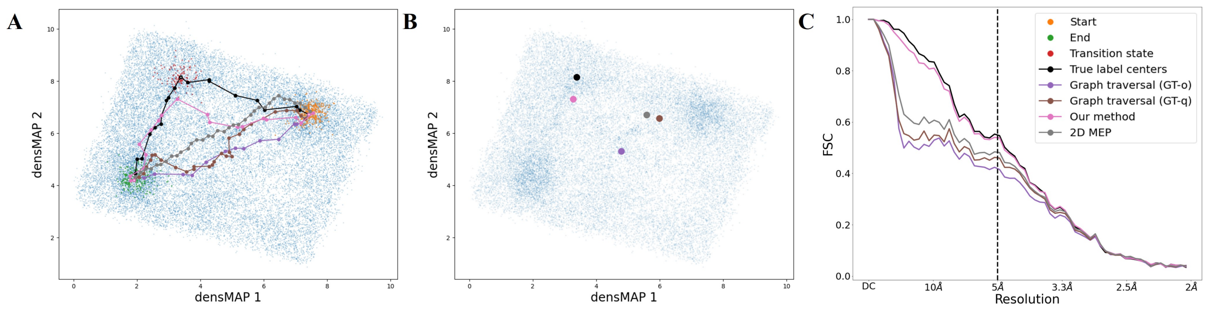

Supplementary Fig. 6: Results of the subset from the Hsp90 experiment that contains 30,000 images. **A**: Paths from various algorithms in the densMAP 2D space are displayed, with orange, green, and red dots representing starting, ending, and transition states, respectively. The ground truth trajectory is depicted as a black line. Paths are color-coded: GT-o in purple, GT-q in brown, 2D MEP in gray, and our energy-aware method in pink. **B**: A 2D visualization illustrates each path's nearest point to the ground truth transition state in the latent space. **C**: FSC curves depict the similarity between the 3D volume of each algorithm and the ground truth transition state volume.

Table 3. Experimental results for various metrics in the subset from the Hsp90 dataset, which contains 10,000 images, are presented. In this analysis, we report the mean and standard deviation of the metrics over five independent runs. The algorithms compared include GT-o, GT-q, 2D MEP, and the energy-aware pathfinding algorithm. Here, g_i signifies the center of state i in the latent space, while P and G denote the sequence of points on the predicted path and the center points of the states on the ground truth path, respectively.

Hsp90	GT-o	GT-q	2D MEP	Our energy-aware method
$FSC_{5\text{Å}}(g_{Transition}, P)$	0.397 (0.033)	0.400 (0.042)	0.411 (0.021)	**0.438** (0.033)
$d_{\text{Transition}}(g_{Transition}, P)$	3.479 (0.919)	2.943 (1.969)	2.824 (1.036)	**1.276** (1.325)
$D_{\text{ave}}(G, P)$	2.182 (0.576)	1.916 (1.048)	2.084 (0.633)	**0.965** (0.562)
$D_{\text{worst}}(G, P)$	3.551 (0.891)	3.254 (1.463)	4.356 (0.265)	**1.699** (0.994)

H. The robustness of the framework when dealing with smaller datasets

Due to the inherent complexity of real-world datasets, the latent space learned might not always sufficiently capture structural heterogeneity, potentially leading to suboptimal outcomes during the search phase of our pathfinding algorithm. To evaluate the limitations of our framework under non-ideal conditions, we conducted an experiment by randomly sampling 30,000 images from the Hsp90 dataset, with five independent runs, and repeating the original experiment.

The resulting space from one run is depicted in Supplementary Fig. 6. It became notably more challenging to distinguish the transition state from many others, even when utilizing densMAP, which represents more challenging conditions. Subsequently, we conducted a similar experiment in this space, analogous to our approach with the original Hsp90 dataset. We used the centers of major states in the latent space as inputs for the pathfinding algorithms and established the ground truth trajectory by connecting the centers of the transition states. The results are presented in Supplementary Fig. 6A, showing a pattern similar to the original experiment, indicating that the energy-aware method outperforms others.

For quantitative comparison, we calculated each metric over five independent runs and reported the mean and variance, as shown in Supplementary Table 2. When calculating $FSC_{5\text{Å}}$, our energy-aware method yielded top scores of 0.534, closely mirroring the score from using the 3D volume from the center of the transition state, as shown in Supplementary Table 2. The $d_{\text{Transition}}$ metric reflects a similar pattern, with the path identified by our methodology being the nearest to the transition state located in the top-right corner. Moreover, according to the overall distance metric, our method exhibited the closest proximity to the ground truth path, registering an average distance of 0.660 as represented by D_{ave} and a value of 1.316 in terms of D_{worst}, illustrating that our energy-aware pathfinding method consistently outperforms others in this smaller dataset. These results suggest that our proposed method remains robust even when the underlying distribution of occupancy deviates from ideal cases.

To further test the limitations of our algorithm, we conducted an experiment by randomly sampling 10,000 images from the Hsp90 dataset with five independent runs and repeating the original experiment. Again, we calculated each metric over five independent runs and reported the mean and variance, as shown in Supplementary Table 3. Our energy-aware algorithm still significantly outperformed the other methods. However, the variation in the retrieved path increased, indicating that the retrieved pathway still depends on the recovered latent space. Therefore, if the underlying distribution of occupancy deviates significantly from the true distribution, all subsequent searches will fail. This scenario can occur in situations like an insufficient amount of particles collected, as in this experiment, or failure to capture short-lived transition states during the data collection stage. In summary, our energy-aware method can robustly retrieve the underlying pathway when the underlying distribution of occupancy deviates from ideal cases by a moderate amount.

Table 4. Experimental results of the 3DVA latent space analysis for the Hsp90 and NLRP3 datasets: This study compares several algorithms, including GT-o, GT-q, 2D MEP, and the energy-aware pathfinding algorithm. The symbol g_i denotes the center of state i in the latent space. Additionally, P represents the sequence of points along the predicted path, and G indicates the center points of the states on the ground truth path, respectively.

Hsp90	GT-o	GT-q	2D MEP	Our energy-aware method
$FSC_{5\text{Å}}(g_{Transition}, P)$	0.576	0.541	0.566	**0.770**
$d_{\text{Transition}}(g_{Transition}, P)$	154.975	162.533	156.070	**33.889**
$D_{\text{ave}}(G, P)$	99.257	91.233	117.899	**65.191**
$D_{\text{worst}}(G, P)$	**154.975**	168.848	181.690	179.510
**NLRP3**	**GT-o**	**GT-q**	**2D MEP**	**Our method**
$FSC_{5\text{Å}}(g_{Transition}, P)$	0.476	0.476	0.462	**0.631**
$d_{\text{Transition}}(g_{Transition}, P)$	139.056	139.056	142.946	**20.030**
$D_{\text{ave}}(G, P)$	96.380	93.908	102.649	**29.373**
$D_{\text{worst}}(G, P)$	139.934	139.934	196.503	**45.964**

I. The robustness of the framework when dealing with latent space recovered by 3DVA

As discussed in the main text, *CLEAPA* assumes the recovered conformation landscape to be sufficiently accurate. Our choice of cryoDRGN as the reconstruction model is based on its ability to learn an accurate conformational landscape from both synthetic and real datasets, as detailed in Appendix E. In this section, we examine the performance of the pathfinding algorithm when there is a moderate deviation in the recovered landscape from the actual occupancy map. Specifically, we investigate the conformation landscape

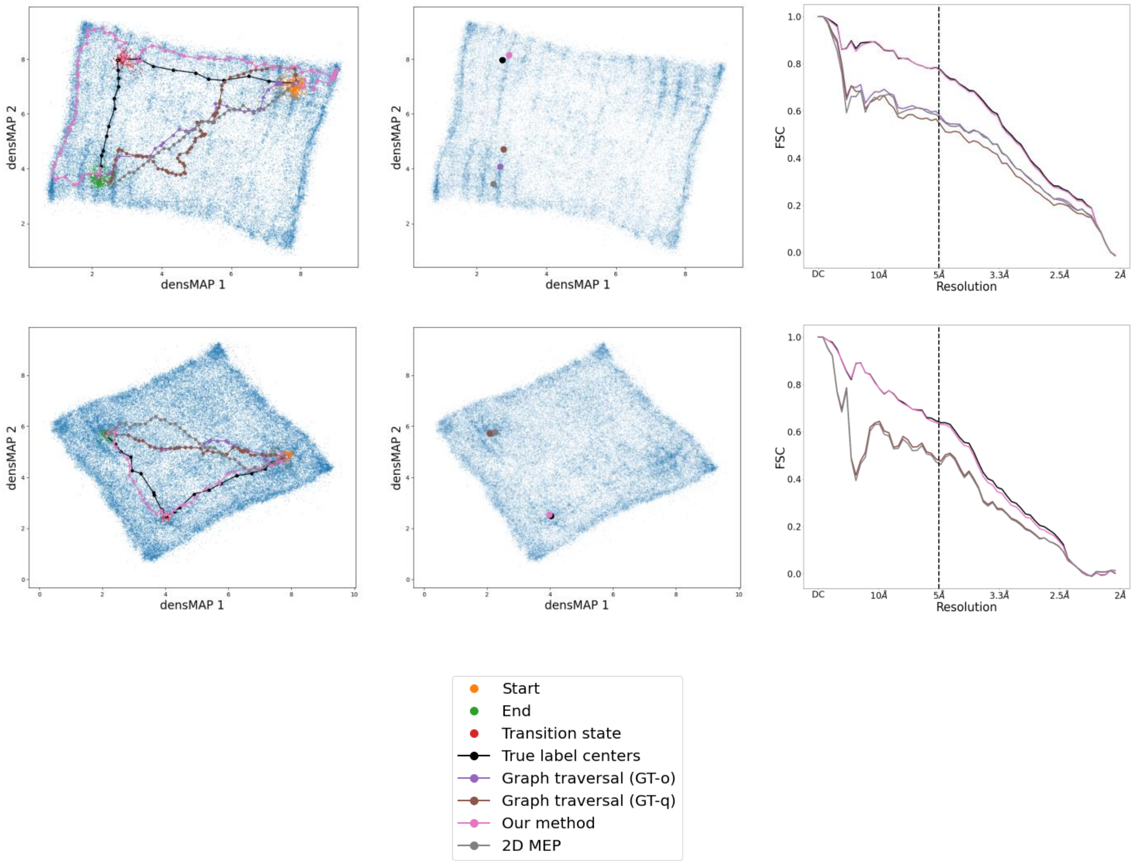

Supplementary Fig. 7: Results of 3DVA on the Hsp90 and NLRP3 datasets: The first row pertains to Hsp90, and the second row to NLRP3. In the first column, paths from various algorithms in the densMAP 2D space are illustrated, where orange, green, and red dots indicate the starting, ending, and transition states, respectively, against the ground truth trajectory shown as a black line. The paths are color-coded as follows: GT-o in purple, GT-q in brown, 2D MEP in gray, and our energy-aware method in pink. The second column presents a 2D visualization of each path's nearest point to the ground truth transition state in the latent space. The third column displays the FSC curves, reflecting the similarity between the 3D volume of each algorithm's path and the ground truth transition state volume.

recovered by 3DVA Punjani and Fleet (2021a), which, according to Dsouza et al. (2023), ranks just below cryoDRGN in accuracy but is also widely used in the field due to its lesser need for hyperparameter tuning and efficient extraction of conformational landscapes with dimensionality exceeding two.

We adhere to the same data preparation steps described in Appendix F. For 3DVA's key hyperparameter, the number of modes is set to 8, producing an eight-dimensional latent space akin to that of cryoDRGN, and the filter resolution is set to 5 Å, a commonly used value. The resulting landscapes for Hsp90 and NLRP3 are depicted in Supplementary Fig. 7. At first glance, both the main and transition states are preserved as high-density regions within the recovered landscape, with the behavior of the pathfinding algorithms mirroring that seen with cryoDRGN: the energy-aware pathfinding algorithm adheres to the ground truth path, while other algorithms tend to cross the middle region, missing the transition state. However, further inspection reveals notable deviations from the ideal case, particularly some unusually high-density areas located on the borders rather than along the ground truth path. This deviation causes our energy-aware pathfinding algorithm to identify a path toward these border regions. The situation deteriorates with the Hsp90 dataset; despite accurately capturing the transition state, it identifies a border path, evidently a low-energy path in the recovered landscape.

Quantitatively, for the Hsp90 dataset, the ground truth 3D volume centered on the transition state yields an $FSC_{5\text{Å}}$ of 0.774, with our method showing a similar correlation at 0.770, as detailed in Supplementary Table 4. Additionally, the $d_{\text{Transition}}$ metric confirms our predicted path's proximity to the target ground truth transition state. The path identified by our method remains closest to the ground truth path per the D_{ave} metric, though the maximum discrepancy, according to D_{worst}, is 179.510, higher than GT-o and GT-q due to an unusual high-density region at the border. For the NLRP3 dataset, the ground truth 3D volume centered on the transition state yields an $FSC_{5\text{Å}}$ of 0.638, with our energy-aware method showing a similar correlation at 0.631. All other metrics indicate that

the energy-aware method significantly surpasses other pathfinding algorithms. These findings reinforce the robustness of our proposed method even when the underlying occupancy distribution deviates from ideal scenarios.

To further test the limitations of our algorithm, we conducted an experiment on the latent space recovered from the EMPIAR-10076 real dataset, with results shown in Supplementary Fig. 8. In this instance, the conformational landscape is distorted, unable to distinguish the minor states in the C, D, and E regions. Moreover, sparse regions located between the four main states complicate the task for the pathfinding algorithm, which tends to traverse directly over the middle region, failing to adhere to the published path, as illustrated in Supplementary Fig. 8B. The primary challenge is that the 3DVA algorithm employs a linear subspace model, utilizing subspace components to span a continuous space of possible states. However, the true conformational landscape may reside on a manifold, characterized by non-linear and complex geometry, making accurate recovery by 3DVA challenging.

In conclusion, our experiments suggest that significant deviations from the true occupancy distribution result in the failure of subsequent searches, as evidenced in the EMPIAR-10076 dataset. However, if the deviation is moderate and the states are distinctly separated, as observed in the Hsp90 and NLRP3 datasets, our energy-aware method can still robustly identify the underlying pathway. We are optimistic that with the ongoing development of nonlinear models, their ability to approximate the true distribution of biomolecular structures will improve, thereby enhancing the efficacy of our framework.

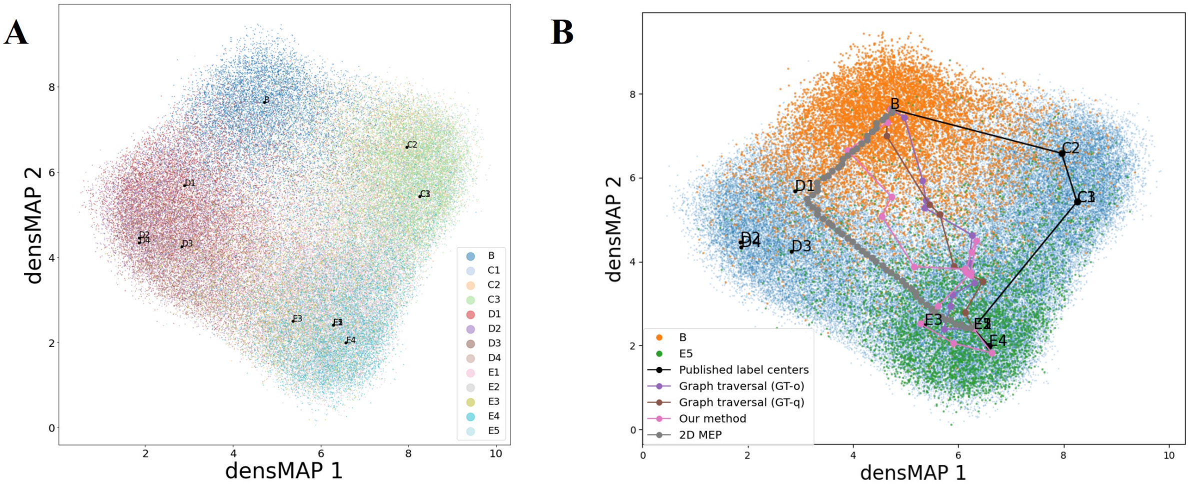

Supplementary Fig. 8: Visualization of the 2D embedding of the latent space for the EMPIAR-10076 dataset via 3DVA and results of pathfinding algorithms. **A**: This panel shows a 2D visualization of the 3DVA latent space utilizing densMAP, where each color denotes a unique conformational state. Black dots represent the median point in latent space for each state. Notably, states labeled A, F, and Discarded are excluded from this visualization. **B**: Displays paths from various algorithms within the densMAP 2D space. Orange, green, and red dots signify the starting, ending, and transition states, respectively, alongside the published trajectory marked by a black line. Path coloration includes GT-o in purple, GT-q in brown, 2D MEP in gray, and our energy-aware method in pink.

J. Enhance the local density estimation using kernel

As discussed in the main text, a potential limitation of our energy-aware pathfinding algorithm manifests in situations where the graph contains a high number of zero-energy edge weights. In such cases, our method may struggle. Specifically, the shortest path might inadvertently select many points from regions with zero edge weights. This behavior can be better understood through visualization of the energy distribution on the conformational landscape, as depicted in Supplementary Fig. 9. From the first column of the figure, it is evident that our algorithm tends to select more points within the main and transition states, resulting in small loops and turns in these regions that extend the path length. These minor loops or turns can generate duplicated or confusing frames during the creation of the final dynamic movie along the trajectory. The underlying cause of this phenomenon becomes apparent in the second and third columns of Supplementary Fig. 9. In the second column, we illustrate each point in the 2D embedding using a color indicative of the energy E_v estimated by our algorithm. It is observable that large regions are occupied by low-energy points either in the main state or the transition state, especially in the EMPIAR 10076 dataset. Consequently, the algorithm may encounter challenges in identifying the shortest path because the energy levels of neighboring nodes are approximately equal, lacking a distinct gradient to guide the search process. In the third column of Supplementary Fig. 9, we present a histogram of the energy estimates E_v. It is clear that there is a significant prevalence of low-energy points, with the proportion in the lowest energy bin nearing 10% in the EMPIAR 10076 dataset. This suggests a rationale for the observed looping or turning patterns within the embedding.

To alleviate the identified issue, we introduce a kernel function to enhance local density estimation. The underlying intuition is that the kernel function can produce differences in the estimation by adjusting the bandwidth, thereby highlighting underlying trends more

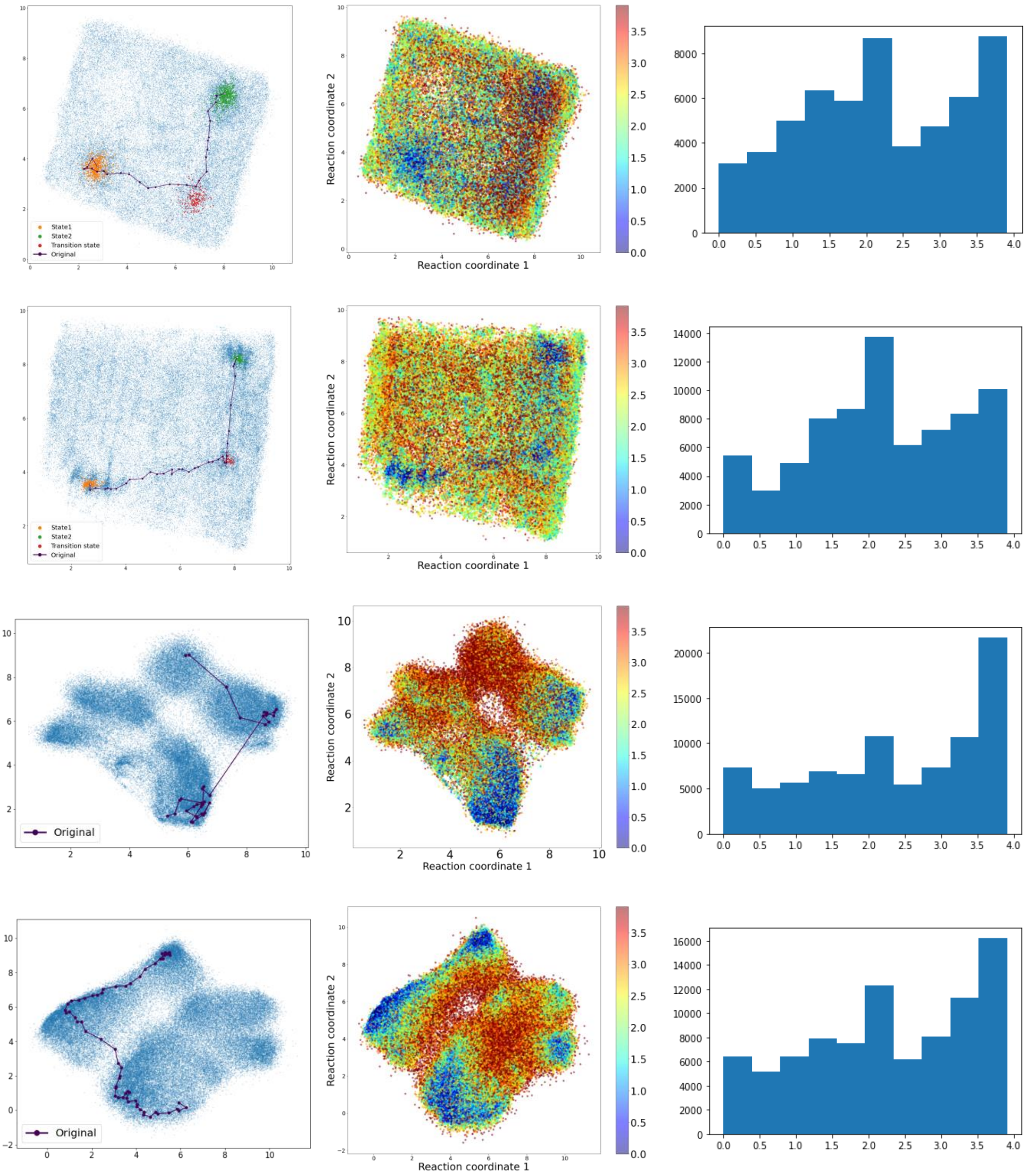

Supplementary Fig. 9: The visualization of paths and statistics derived from our energy-aware pathfinding algorithm with default settings. The display is segmented into columns (1) to (3), from left to right. Column (1) delineates the paths identified by the algorithm within the 2D embedding space across four datasets utilized in this research. Concurrently, column (2) illustrates the 2D embedding of these datasets, each color-coded based on the energy estimations provided by our algorithm at individual data points. Column (3) shows histograms portraying the distribution of energy estimates computed at each data point by our algorithm. Progressing from top to bottom, the rows illustrate results obtained from the Hsp90, NLRP3, EMPIAR-10076, and EMPIAR-10076 after analyze-landscape datasets, respectively.

prominently. Moreover, it offers a more robust estimate, demonstrating resistance to noise and outliers in the dataset. In this study, we have chosen the Gaussian kernel to down-weight the contributions of distant neighbors or outliers. The new local density estimation equation is defined as follows:

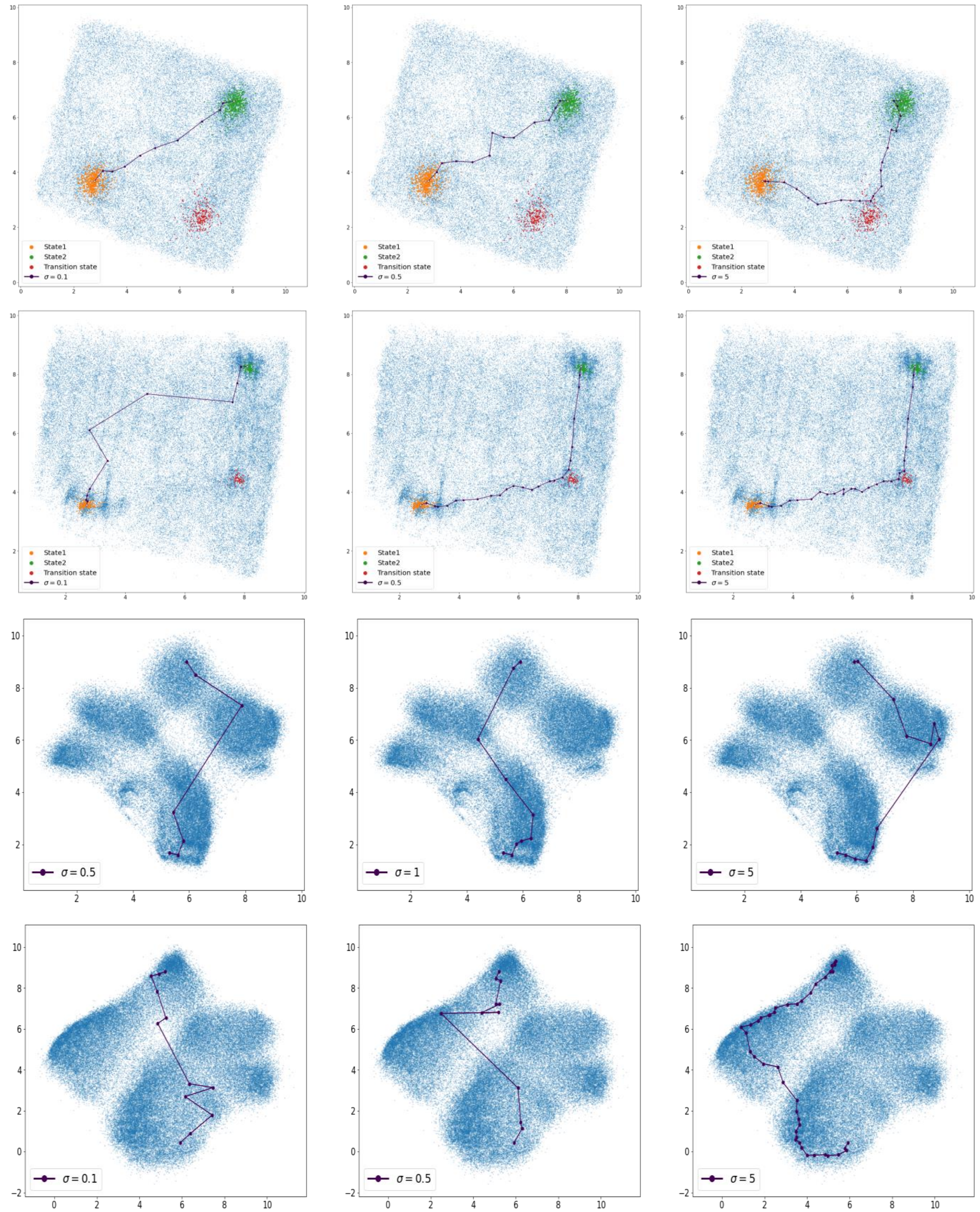

Supplementary Fig. 10: The paths identified by our energy-aware pathfinding algorithm with varying bandwidths. Displayed from left to right are columns (1) to (3). Column (1) shows paths found by the algorithm employing a small bandwidth across four datasets analyzed in this study. Similarly, columns (2) and (3) represent paths established with medium and large bandwidths, respectively. The rows, arranged from top to bottom, depict the results obtained from the Hsp90, NLRP3, EMPIAR-10076, and EMPIAR-10076 after analyze-landscape datasets, respectively.

$$n'_v = \sum_{i=1}^{N'} \exp(\frac{-(v - v_i)^2}{2\sigma^2}) \tag{S.6}$$

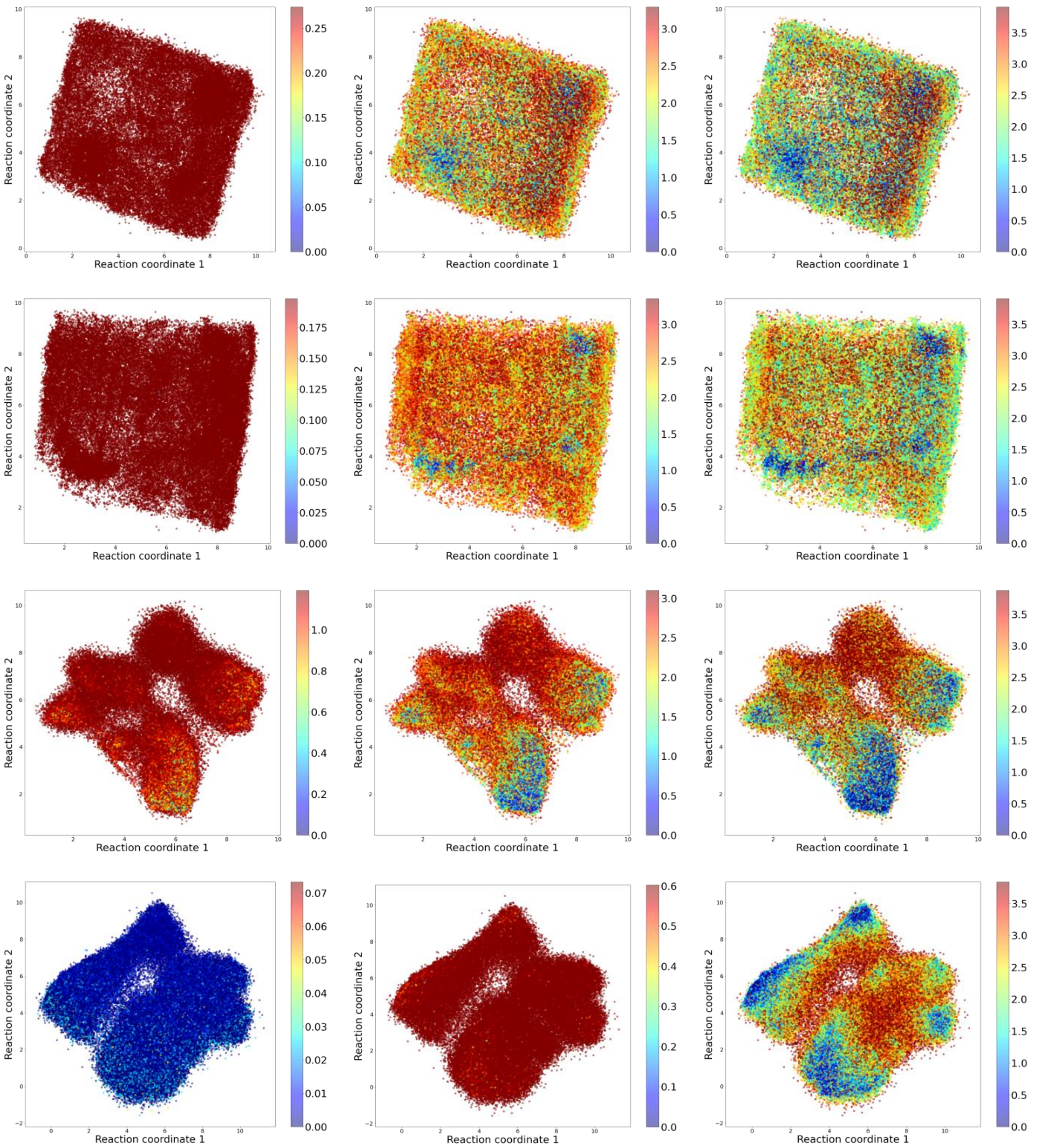

Supplementary Fig. 11: The 2D embedding space is depicted with varying color intensities to represent the energy estimations by our energy-aware pathfinding algorithm at each data point across different bandwidths. The figure is structured from left to right into columns (1) to (3). Column (1) illustrates the energy estimations in the 2D embedding space with a small bandwidth applied to four datasets analyzed in this study; Column (2) highlights the energy estimations with a medium bandwidth; Column (3) depicts the energy estimations with a large bandwidth. Sequentially from top to bottom, the rows represent the results derived from the Hsp90, NLRP3, EMPIAR-10076, and EMPIAR-10076 after analyze-landscape datasets, respectively.

where N' represents the number of neighbors for each node, determined after pruning with the threshold θ, and σ denotes the bandwidth of the kernel. Upon determining σ, the local density n'_v can be calculated, and the new energy estimation E'_v for each node can be obtained using Equation (S.2). It is noteworthy that this new statistic extends the original local density estimation approach; as $\sigma \to \infty$, the estimation converges to the original approach presented in the main text. Furthermore, by reducing σ, the number of low-energy points decreases, which tends to provide clearer guidance for the pathfinding algorithm in seeking a shorter path.

To evaluate the influence of the kernel function, we selected three different bandwidths to determine the path and energy statistics for each bandwidth across four datasets. The results are depicted in Supplementary Figs. 10 to 12. As observed from the figures, a decrease in bandwidth from right to left encourages the pathfinding algorithm to identify a route of shorter length, as an increasing number of points exhibit high and approximately equal energy levels. Moreover, regions with a substantial concentration of low-energy points begin to display more pronounced energy level disparities, thereby furnishing a guide for the pathfinding algorithm to avoid unnecessary loops and turns. Empirically, we ascertain that a setting of $\sigma = 5$ enables the selection of a path that resembles the original but without the inclusion of unwarranted loops or turns. A comparison of metrics between this configuration and the default setting is summarized in Supplementary Table 5. Notably, while most metrics align closely with those of the original configuration, the D_{worst} metric consistently surpasses the default setting. This implies that the path identified by the new configuration is likely to produce a more realistic dynamic movie portraying conformational changes, effectively excluding outlier frames.

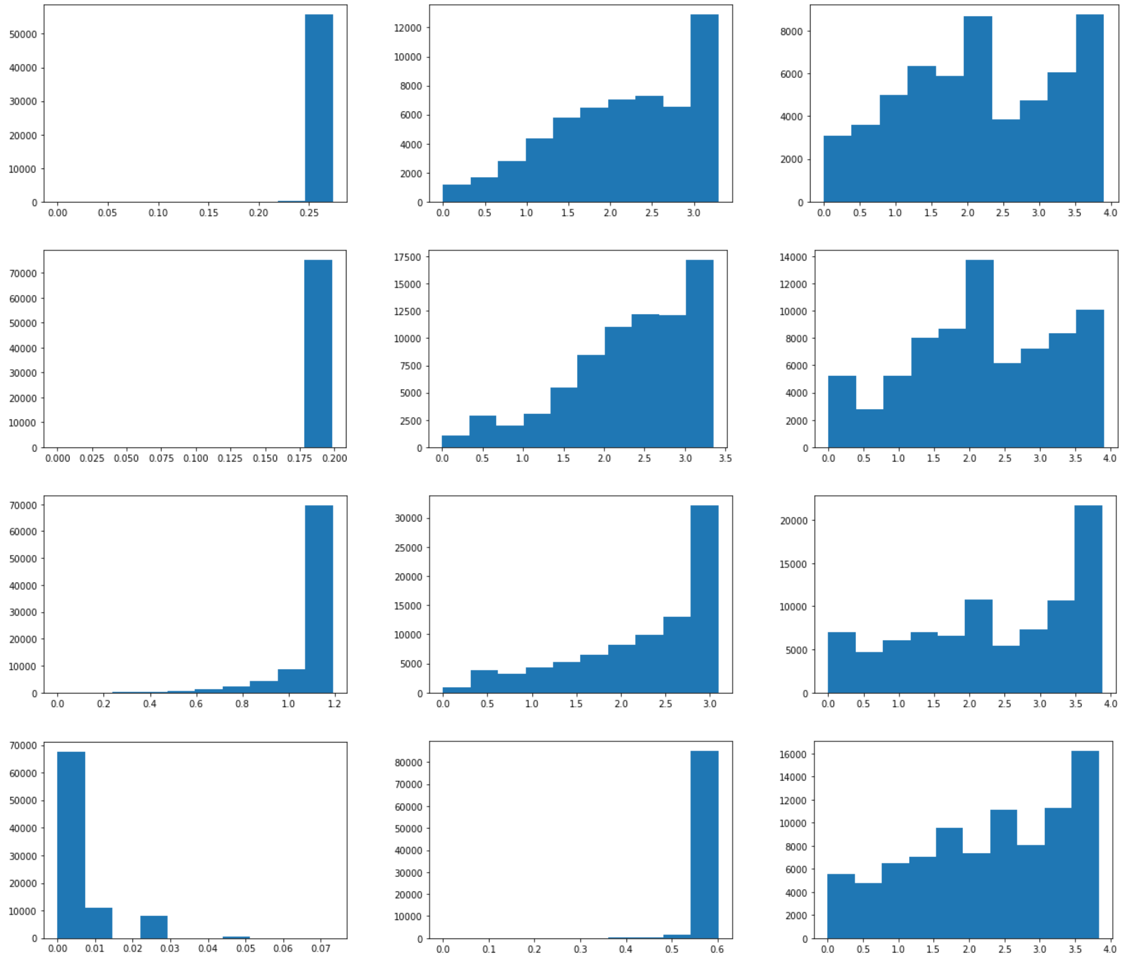

Supplementary Fig. 12: The figure displays histograms representing energy estimations made by our energy-aware pathfinding algorithm at each data point, utilizing different bandwidths. Spanning from left to right are columns (1) to (3). Column (1) presents the histogram of energy estimations conducted with a small bandwidth across four datasets analyzed in this study. Similarly, columns (2) and (3) depict the histograms of energy estimations achieved with medium and large bandwidths, respectively. The rows are organized from top to bottom to exhibit results obtained from the analyses of the Hsp90, NLRP3, EMPIAR-10076, and EMPIAR-10076 after analyze-landscape datasets, respectively.

Upon analyzing the impact on each dataset, we observe two potential paths emerging when the bandwidth is modified within the Hsp90 and NLRP3 datasets. One option is a path that traverses through the transition state, while the alternative offers a direct connection between the start and end states. It becomes evident that a reduction in bandwidth encourages the algorithm to select a shorter path that manages to overcome some high-energy barriers on the landscape. In the case of the EMPIAR-10076 dataset, we notice that a

Table 5. Experimental results for different metrics across all datasets. The algorithms compared are the default energy-aware pathfinding algorithm and the energy-aware pathfinding algorithm with kernel function ($\sigma = 5$). g_i signifies the state's center i in the latent space, while P and G denote the sequence of points on the predicted path and the center points of the state on the ground truth path, respectively.

**Hsp90**	**default**	$\sigma = 5$
$FSC_{5Å}(g_{Transition}, P)$	0.578	0.575
$d_{\text{Transition}}(g_{Transition}, P)$	0.897	0.948
$D_{\text{ave}}(G, P)$	0.628	0.648
$D_{\text{worst}}(G, P)$	1.214	1.138
**NLRP3**	**default**	$\sigma = 5$
$FSC_{5Å}(g_{Transition}, P)$	0.485	0.494
$d_{\text{Transition}}(g_{Transition}, P)$	0.602	0.601
$D_{\text{ave}}(G, P)$	0.587	0.484
$D_{\text{worst}}(G, P)$	1.154	1.033
**EMPIAR-10076**	**default**	$\sigma = 5$
$FSC_{5Å}(g_{C2}, P)$	0.942	0.942
$FSC_{5Å}(g_{E2}, P)$	0.993	0.963
$FSC_{5Å}(g_{E4}, P)$	0.989	0.961
$d_{\text{Transition}}(g_{C2}, P)$	1.915	1.915
$d_{\text{Transition}}(g_{E2}, P)$	0.769	2.046
$d_{\text{Transition}}(g_{E4}, P)$	1.107	1.287
$D_{\text{ave}}(G, P)$	1.162	1.524
$D_{\text{worst}}(G, P)$	3.214	2.374
**EMPIAR-10076 after analyze-landscape**	**default**	$\sigma = 5$
$FSC_{5Å}(g_{C2}, P)$	0.894	0.884
$FSC_{5Å}(g_{E2}, P)$	0.962	0.963
$FSC_{5Å}(g_{E4}, P)$	0.932	0.924
$d_{\text{Transition}}(g_{C2}, P)$	2.175	2.680
$d_{\text{Transition}}(g_{E2}, P)$	1.787	1.744
$d_{\text{Transition}}(g_{E4}, P)$	2.318	2.090
$D_{\text{ave}}(G, P)$	2.427	2.504
$D_{\text{worst}}(G, P)$	8.525	7.763

larger value of σ facilitates the identification of the longest conformation change path, represented by the blue path in Supplementary Fig. 20. Conversely, a decrease in σ tends the algorithm towards the orange path, indicative of a shorter conformation change route. Given the intricate nature of the underlying system, we believe that the flexibility introduced by implementing the kernel function with varying bandwidths will enable us to investigate a broader spectrum of possible transition pathways. For example, the functional pathway between two states might be more accurately represented by a path that not only minimizes cumulative energy but also overcomes the lowest maximum energy peak Seitz and Frank (2020). Our standard local density estimation n_v typically identifies a minimum energy path that achieves this criterion. However, by decreasing σ in n'_v, it becomes possible to explore paths that, while maintaining similar integrated energy levels, might traverse some moderate peaks, thereby revealing additional potential competing low-energy pathways. It is noted that the visualizations presented in Supplementary Figs. 11 and 12 significantly aid users in selecting an appropriate landscape by examining the energy distribution.

In conclusion, the integration of a kernel function into our algorithm presents several advantages. Primarily, it offers more explicit guidance to the algorithm, effectively avoiding unnecessary detours. Secondly, it improves resilience to outliers and noise by diminishing the influence of distant neighbors, thus enabling a more precise and robust estimation of local density. Lastly, this strategy, coupled with visualizations, paves the way for investigating various conformational transition pathways within complex systems, a capability prominently showcased in the analysis of the EMPIAR-10076 dataset.

K. Runtime Comparison of Different Pathfinding Algorithms

To assess the computational efficiency of various pathfinding algorithms, we executed each algorithm 10 times to calculate the average computation time and standard deviation, as detailed in Supplementary Table 6. Performance measurement was conducted using Python's standard benchmark module, `timeit`. For the 2D MEP, the time spent by POLARIS was directly obtained through the POLARIS GUI and added to the densMAP to determine the total runtime. The experiments were performed on a laptop featuring an AMD Ryzen 5 3600 6-core processor, 32 GB of RAM, and an NVIDIA GeForce RTX 3080 GPU.

From Supplementary Table 6, it is clear that our energy-aware method outperforms both GT-q and 2D MEP in terms of speed but trails behind GT-o. The comparative slowness against GT-o stems from the necessity to estimate local density for incorporating energy into our pathfinding algorithm and employing a quantile search algorithm (Appendix D) to find the optimal threshold for local density estimation. Despite this, the significant performance improvement, as elaborated in the main text, justifies the additional computational efforts. Moreover, our method obviates the need for running Dijkstra's Algorithm at each threshold (quantile), unlike GT-q, by sidestepping quantiles that fall outside the predefined zero energy ratio range (typically set between 0.01 to 0.1). This approach not

only ensures the identification of suitable landscapes but also markedly reduces computational time compared to GT-q. It should be noted that the 2D MEP might demand more time in practice, as users may need to conduct multiple densMAP runs with parameter adjustments to identify the most appropriate 2D energy landscape. This necessity makes it somewhat less efficient compared to graph-based searching methods that probe directly in latent space.

In summary, all the algorithms within our framework are capable of completing the search process in just a few minutes for datasets of a typical size on a laptop, a stark contrast to traditional methods that might take days to weeks to determine the path through several rounds of 3D classifications Davis et al. (2016). This efficiency highlights the significant potential of pathfinding algorithms in the realm of protein structure research.

Table 6. Comparison of runtime performance for various pathfinding algorithms: Detailed analysis of mean and standard deviation across 10 iterations, with experimental parameters aligned with those specified in the main text, measured in seconds.

	**GT-o**	**GT-q**	**2D MEP**	**Our energy-aware method**
**Hsp90**	6.979 (0.072)	59.187 (0.143)	87.947 (2.430)	54.033 (1.420)
**NLRP3**	9.786 (0.373)	78.070 (1.333)	117.473 (0.691)	72.184 (1.277)
**EMPIAR-10076**	11.197 (0.663)	89.698 (1.304)	176.681 (5.416)	40.260 (0.750)

L. Supplementary Figures

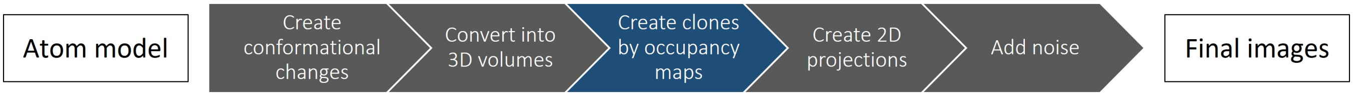

Supplementary Fig. 13: Flowchart of the workflow for the generation of the synthetic dataset with continuous conformational changes

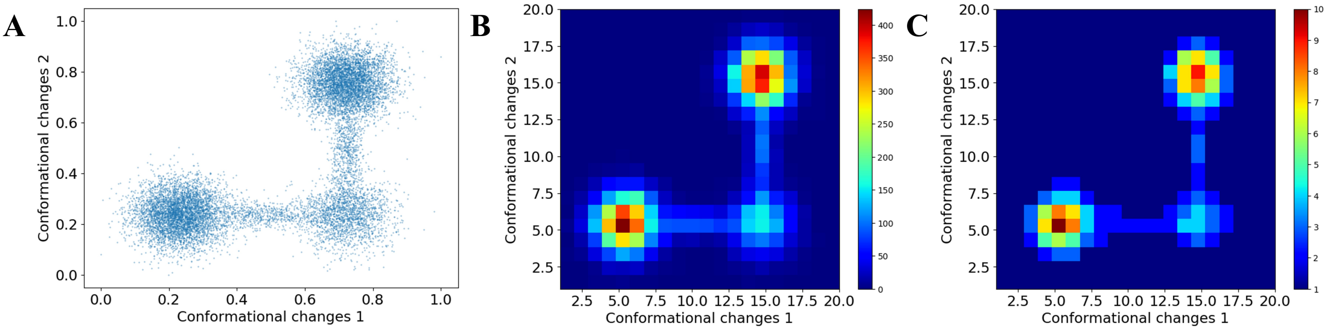

Supplementary Fig. 14: Illustration of the occupancy map generation process with a 2-degree conformational change and 20 conformational states for each coordinate. **A**: Data points are generated using a Gaussian mixture model in a 2D space, embodying the conformational changes with two degrees of freedom. The data point distribution is designed to emulate the presence of different conformational states in the dataset. **B**: Upon segmenting the coordinates into 20 bins, the number of data points within each grid is counted. These counts denote the frequency of each conformational state, with a higher count signifying a more stable or commonly observed state. **C**: To optimize computational efficiency, the counts are rescaled to the desired occupancies, representing the number of clones for each conformational state.

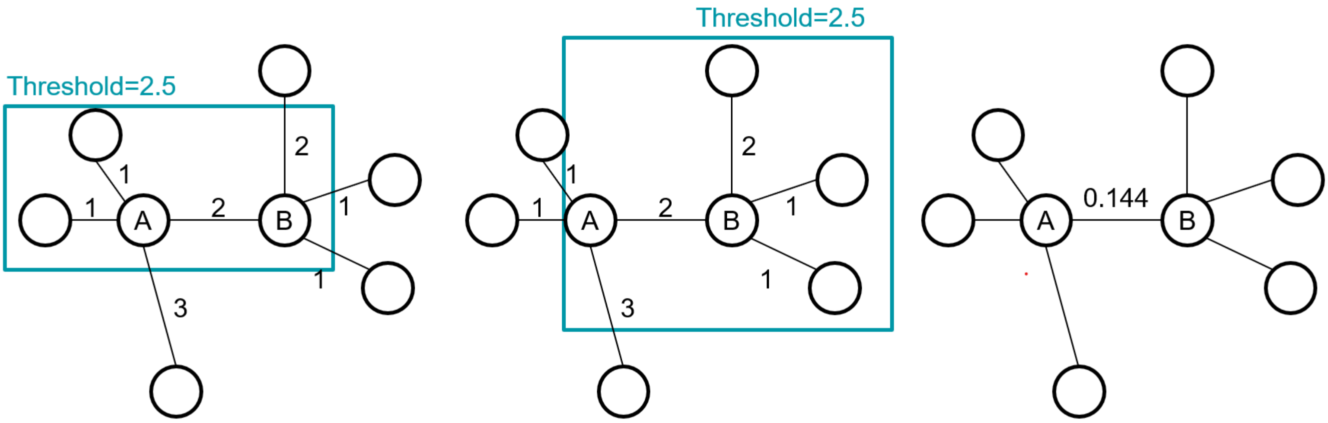

Supplementary Fig. 15: Example of energy edge weighting scheme for a given threshold. The example graph is constructed using 4 nearest neighbors. A predetermined threshold of 2.5 prunes the graph, and node A retains 3 neighbors within this threshold. The energy for node A is computed as 0.288 using Equation (S.2). Node B maintains 4 neighbors within the threshold, representing the graph's highest occupancy, thus resulting in an energy of 0. Finally, the edge weights between nodes A and B are computed as the two nodes' average energy, which is 0.144.

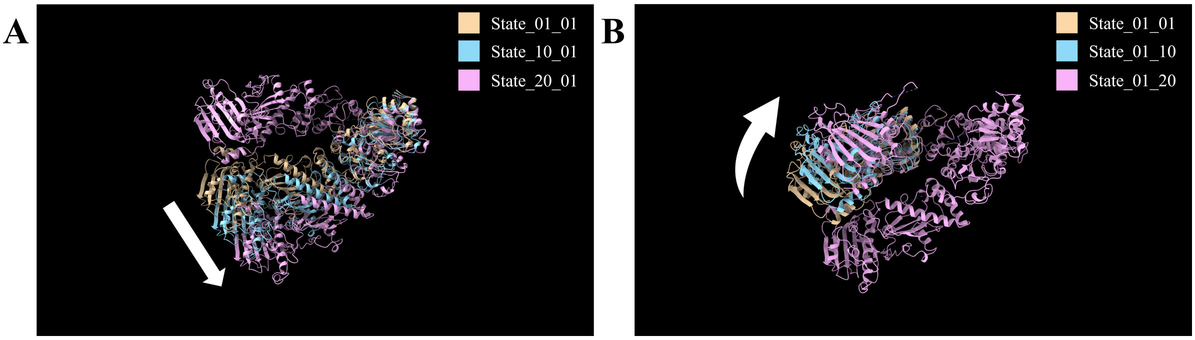

Supplementary Fig. 16: Illustration of conformational changes in the Hsp90 experiment. **A**: Conformational changes in the first direction are modeled by a downward movement of one arm, with a shift of 1 degree for each conformational state. **B**: Conformational changes in the second direction are modeled by an upward rotation of a different arm, with an angular change of 2 degrees for each conformational state.

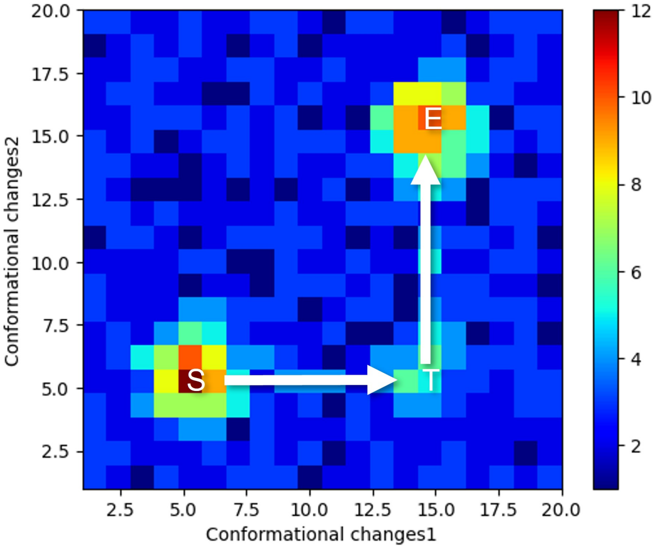

Supplementary Fig. 17: Occupancy map for the Hsp90 experiment. The group at the lower-left corner and the group at the upper-right corner represent the starting (S) and ending (E) conformational states, respectively. The group located at the lower-right corner corresponds to the main transition state (T) we aim to uncover. The occupancy in the background regions is set to 1, and we've randomly added 400 points across the entire map. Paths have been included to provide a clear visual guide for the transition processes.

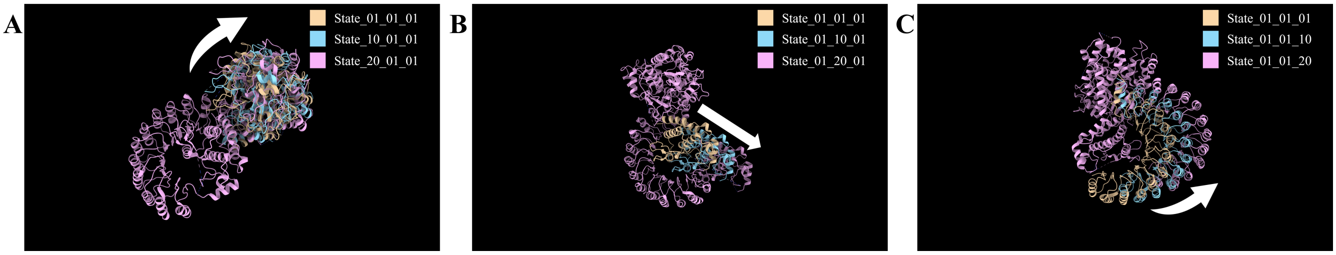

Supplementary Fig. 18: Illustration of conformational changes in the NLRP3 experiment. **A**: The first direction of conformational changes is demonstrated, where the head of the NLRP3 molecule is rotated upward by 2 degrees for each conformational state. **B**: The second direction of conformational changes involves the movement of the binding site by 2 degrees for each conformational state. **C**: The third direction of conformational changes consists of moving the arm upward in the NLRP3 molecule by 2 degrees for each conformational state.

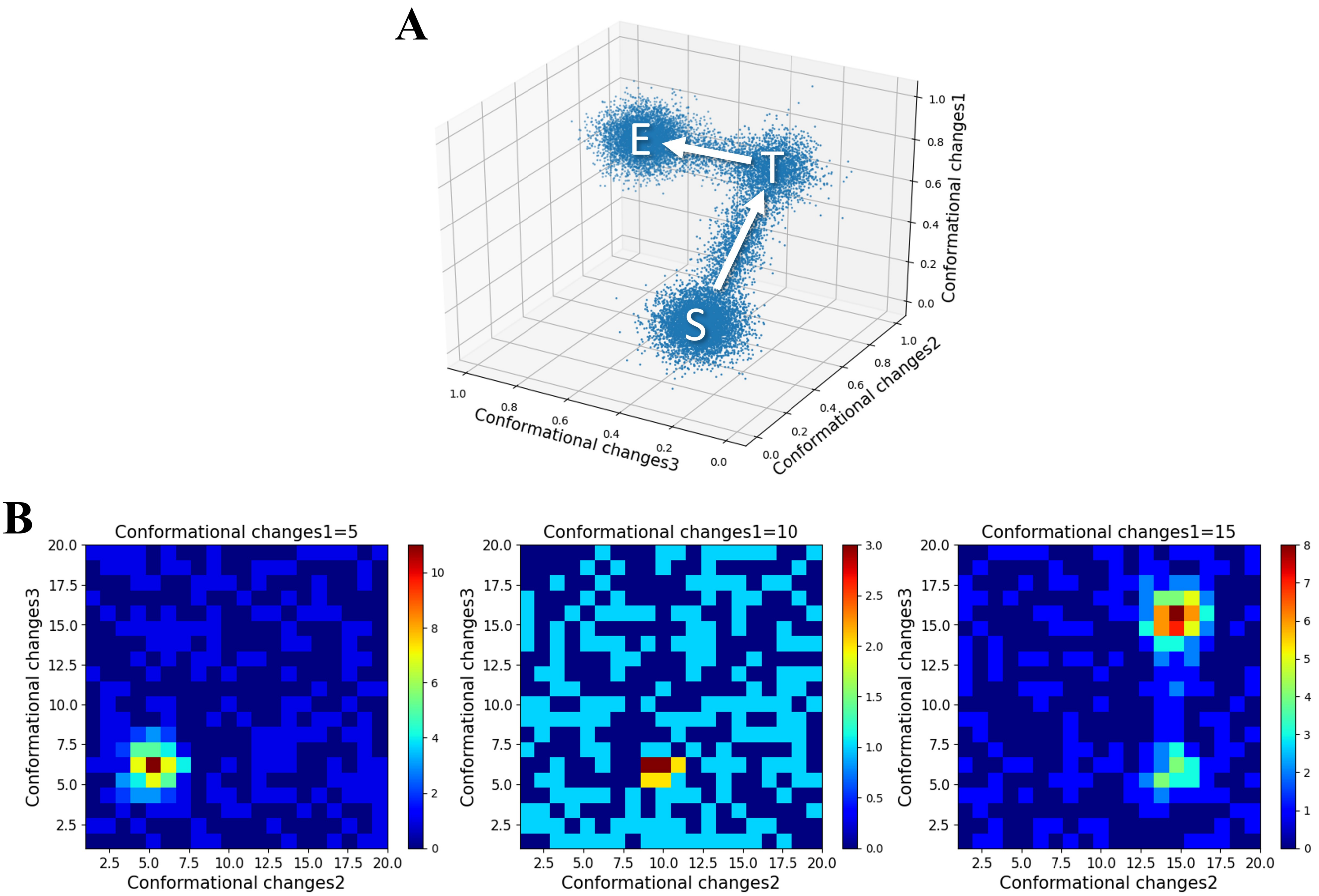

Supplementary Fig. 19: Occupancy map for NLRP3 experiment. **A**: Data points are generated in a 3D space to form the occupancy map, utilizing the same design principle as in the Hsp90 experiment. The two corners denote the starting (S) and ending (E) states, respectively, with the primary transition state (T) situated at another corner. A path links these three groups in the occupancy map. **B**: Slices of the occupancy map along the axis of conformational change 1, with values equal to 5, 10, and 15. Paths have been included to provide a clear visual guide for the transition processes.

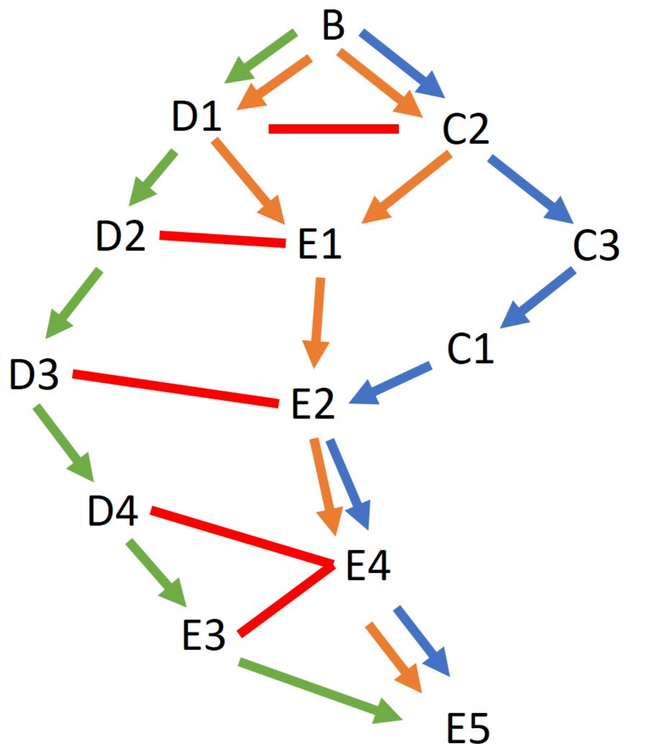

Supplementary Fig. 20: The three conformation change paths suggested in the prior research are visualized in different colors. The first path, consisting of D1, D2, D3, D4, E3, is marked in green. The second path, formed by D1 (or C2), E1, E2, E4, is represented in orange. The third path, incorporating C2, C3, C1, E2, E4, is depicted in blue. Unfavorable transitions are indicated by red lines.

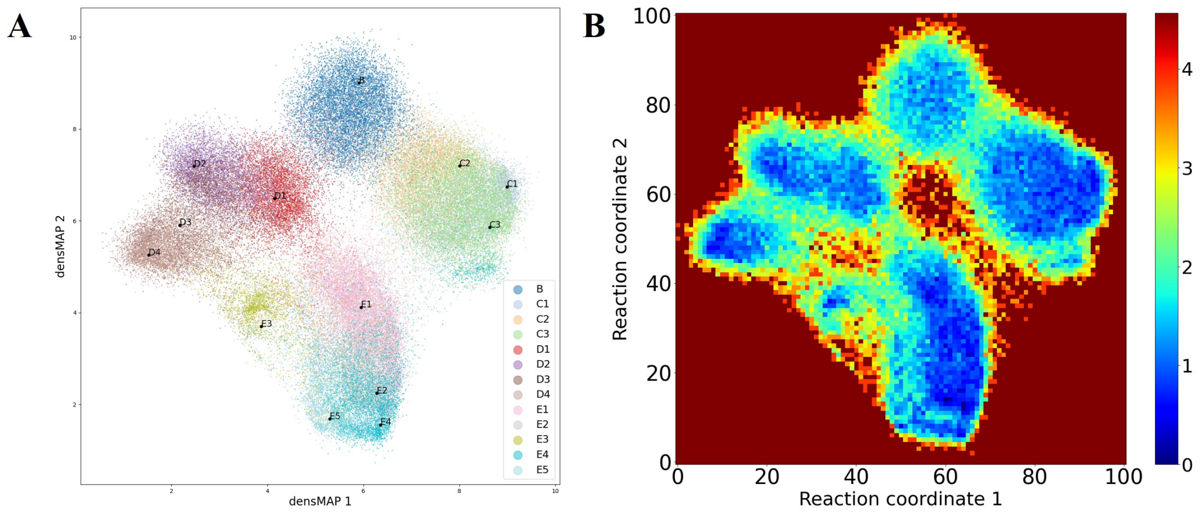

Supplementary Fig. 21: Visualization of the 2D embedding of the latent space and corresponding 2D conformational landscape for the EMPIAR-10076 dataset. **A** 2D densMAP visualization of the latent space for the EMPIAR-10076 dataset. Each color represents a distinct conformational state, with the median point in the latent space for each state indicated by black dots. The states labeled A, F, and Discarded are omitted from this visualization. Furthermore, the C4 state, as identified by the cryoDRGN team, is also excluded. **B** The resulting 2D conformational landscape derived from applying a 2D histogram to the embedding in **A**. Each coordinate is segmented into 100 bins for occupancy count, with energy estimation calculated using Equation (S.2).

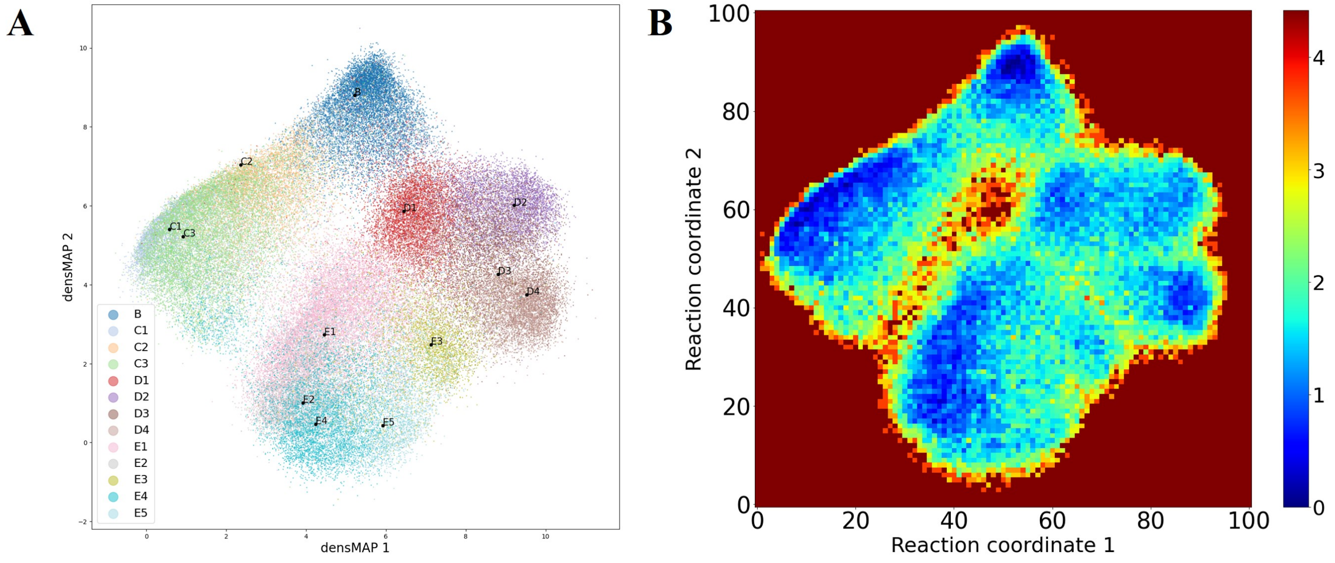

Supplementary Fig. 22: Visualization of the 2D embedding of the latent space for the EMPIAR-10076 Dataset after the analyze-landscape pipeline and corresponding 2D conformational landscape. **A**: 2D visualization of the transformed latent space after the analyze-landscape pipeline, using densMAP. Each color represents a unique conformational state, with black dots indicating the median point in this new space for each state. The states labeled A, F, and Discarded are omitted from this visualization. **B**: The derived 2D conformational landscape, resulting from the application of a 2D histogram to the embedding in **A**. Each coordinate is divided into 100 bins for occupancy counting, with energy estimation computed using Equation (S.2).

References

M. M. Ali, S. M. Roe, C. K. Vaughan, P. Meyer, B. Panaretou, P. W. Piper, C. Prodromou, and L. H. Pearl. Crystal structure of an Hsp90–nucleotide–p23/Sba1 closed chaperone complex. *Nature*, 440(7087):1013–1017, 2006.

T. H. Cormen, C. E. Leiserson, R. L. Rivest, and C. Stein. *Introduction to Algorithms*. MIT press, 2022.

J. H. Davis, Y. Z. Tan, B. Carragher, C. S. Potter, D. Lyumkis, and J. R. Williamson. Modular assembly of the bacterial large ribosomal subunit. *Cell*, 167(6):1610–1622, 2016.

R. Dsouza, G. Mashayekhi, R. Etemadpour, P. Schwander, and A. Ourmazd. Energy landscapes from cryo-EM snapshots: a benchmarking study. *Scientific Reports*, 13(1):1372, 2023.

N. Fischer, A. L. Konevega, W. Wintermeyer, M. V. Rodnina, and H. Stark. Ribosome dynamics and tRNA movement by time-resolved electron cryomicroscopy. *Nature*, 466(7304):329–333, 2010.

L. F. Kinman, B. M. Powell, E. D. Zhong, B. Berger, and J. H. Davis. Uncovering structural ensembles from single-particle cryo-EM data using cryoDRGN. *Nature Protocols*, 18(2):319–339, 2023.

I. Marcos-Alcalde, J. Setoain, J. I. Mendieta-Moreno, J. Mendieta, and P. Gomez-Puertas. MEPSA: minimum energy pathway analysis for energy landscapes. *Bioinformatics*, 31(23):3853–3855, 2015.

I. Marcos-Alcalde, E. López-Viñas, and P. Gómez-Puertas. MEPSAnd: minimum energy path surface analysis over n-dimensional surfaces. *Bioinformatics*, 36(3):956–958, 2020.

L. McInnes, J. Healy, and J. Melville. Umap: Uniform manifold approximation and projection for dimension reduction. *arXiv preprint arXiv:1802.03426*, 2018.

A. Narayan, B. Berger, and H. Cho. Assessing single-cell transcriptomic variability through density-preserving data visualization. *Nature biotechnology*, 39(6):765–774, 2021.

P. A. Penczek, M. Kimmel, and C. M. Spahn. Identifying conformational states of macromolecules by eigen-analysis of resampled cryo-EM images. *Structure*, 19(11):1582–1590, 2011a.

A. Punjani and D. J. Fleet. 3D variability analysis: Resolving continuous flexibility and discrete heterogeneity from single particle cryo-EM. *Journal of structural biology*, 213(2):107702, 2021a.

L. Raff. *Principles of Physical Chemistry*. Prentice Hall, 2001.

S. H. Scheres. A Bayesian view on cryo-EM structure determination. *Journal of molecular biology*, 415(2):406–418, 2012b.

E. Seitz and J. Frank. POLARIS: Path of least action analysis on energy landscapes. *Journal of chemical information and modeling*, 60 (5):2581–2590, 2020.

E. Seitz, F. Acosta-Reyes, S. Maji, P. Schwander, and J. Frank. Recovery of conformational continuum from single-particle cryo-em images: Optimization of manifoldem informed by ground truth. *IEEE transactions on computational imaging*, 8:462–478, 2022.

H. Sharif, L. Wang, W. L. Wang, V. G. Magupalli, L. Andreeva, Q. Qiao, A. V. Hauenstein, Z. Wu, G. Núñez, Y. Mao, et al. Structural mechanism for NEK7-licensed activation of NLRP3 inflammasome. *Nature*, 570(7761):338–343, 2019.

H. D. Tagare, A. Kucukelbir, F. J. Sigworth, H. Wang, and M. Rao. Directly reconstructing principal components of heterogeneous particles from cryo-EM images. *Journal of structural biology*, 191(2):245–262, 2015.

L. Van der Maaten and G. Hinton. Visualizing data using t-SNE. *Journal of machine learning research*, 9(11), 2008.

E. Weinan, W. Ren, and E. Vanden-Eijnden. String method for the study of rare events. *Physical Review B*, 66(5):052301, 2002.

Z. Wu, E. Chen, S. Zhang, Y. Ma, and Y. Mao. Visualizing conformational space of functional biomolecular complexes by deep manifold learning. *International Journal of Molecular Sciences*, 23(16):8872, 2022b.

E. D. Zhong. *Machine Learning for Reconstructing Dynamic Protein Structures from Cryo-EM Images*. PhD thesis, Massachusetts Institute of Technology, 2022.

E. D. Zhong, T. Bepler, J. H. Davis, and B. Berger. Reconstructing continuous distributions of 3D protein structure from cryo-EM images. *arXiv preprint arXiv:1909.05215*, 2019.

E. D. Zhong, T. Bepler, B. Berger, and J. H. Davis. CryoDRGN: reconstruction of heterogeneous cryo-EM structures using neural networks. *Nature methods*, 18(2):176–185, 2021.
